# Development of genomic and genetic resources facilitating molecular genetic studies on untapped Myanmar rice germplasms

**DOI:** 10.1270/jsbbs.23077

**Published:** 2024-03-22

**Authors:** Tomoyuki Furuta, Ohm Mar Saw, Sandar Moe, Khin Thanda Win, Moe Moe Hlaing, Aye Lae Lae Hlaing, Min San Thein, Hideshi Yasui, Motoyuki Ashikari, Atsushi Yoshimura, Yoshiyuki Yamagata

**Affiliations:** 1 Institute of Plant Science and Resources, Okayama University, Kurashiki, Okayama 710-0046, Japan; 2 Department of Agricultural Research, Ministry of Agriculture Livestock and Irrigation, Yezin, Myanmar; 3 Plant Breeding Laboratory, Faculty of Agriculture, Kyushu University, Nishi, Fukuoka 819-0395, Japan; 4 Bioscience and Biotechnology Center, Nagoya University, Furo, Chikusa, Nagoya, Aichi 464-8601, Japan

**Keywords:** genome assembly, genome-wide association study, germplasm, rice, Myanmar

## Abstract

To counteract the growing population and climate changes, resilient varieties adapted to regional environmental changes are required. Landraces are valuable genetic resources for achieving this goal. Recent advances in sequencing technology have enabled national seed/gene banks to share genomic and genetic information from their collections including landraces, promoting the more efficient utilization of germplasms. In this study, we developed genomic and genetic resources for Myanmar rice germplasms. First, we assembled a diversity panel consisting of 250 accessions representing the genetic diversity of Myanmar *indica* varieties, including an elite lowland variety, Inn Ma Yebaw (IMY). Our population genetic analyses illustrated that the diversity panel represented Myanmar *indica* varieties well without any apparent population structure. Second, de novo genome assembly of IMY was conducted. The IMY assembly was constructed by anchoring 2888 contigs, which were assembled from 30× coverage of long reads, into 12 chromosomes. Although many gaps existed in the IMY genome assembly, our quality assessments indicated high completeness in the gene-coding regions, identical to other near-gap-free assemblies. Together with dense variant information, the diversity panel and IMY genome assembly will facilitate deeper genetic research and breeding projects that utilize the untapped Myanmar rice germplasms.

## Introduction

Ongoing population growth and climate change are urgent global issues confronted by plant breeders worldwide (Tester and Langridge 2010). New varieties require resilience to changing local climates and newly emerging insects and diseases (Castañeda-Álvarez *et al.* 2016, Hoisington *et al.* 1999). Therefore, to meet the demand for resilient varieties adapted to local environments, we must evaluate the potential of local and exotic genetic resources.

National seed/gene banks are primary repositories of agriculturally important genetic resources, including many landraces, cultivars, and wild species in each country. Myanmar, the seventh-largest rice-producing country in the world (https://www.fao.org/faostat), has the Seed Bank at the Department of Agricultural Research (DAR) in Yezin. This country is known as a reservoir of unexplored rice genetic resources, including many landraces (Myint *et al.* 2012, Thant *et al.* 2021, Tun *et al.* 2006, Wunna *et al.* 2016, Yamanaka *et al.* 2011). The Seed Bank started collecting and conserving plant genetic resources with 3,000 local rice varieties and currently keeps around 7,000 rice accessions (https://scholars.tari.gov.tw/handle/123456789/1525, Wunna *et al.* 2016). Several studies investigated the genetic structure of those germplasms and revealed that *indica* varieties have been dominantly cultivated in Myanmar (Aye *et al.* 2004, Ohm Mar *et al.* 2006, Shatta *et al.* 1993). Even though the genetic diversity of rice landraces conserved in the Seed Bank in Myanmar was investigated, most of those studies employed simple sequence repeat (SSR) markers for variant detection (Thein *et al.* 2012, Wunna *et al.* 2016, Yamanaka *et al.* 2011). One study reported a genetic diversity analysis using single nucleotide polymorphism (SNP) markers. Diversity array technology (DArT) was applied for SNP detection and identified 7643 SNPs in a collection consisting of 117 samples, which provided a far smaller number of markers than whole-genome resequencing (WGS) in exchange for a sequencing cost (Thant *et al.* 2021). However, no *indica* diversity panel with whole genome variants information has not yet been established from Myanmar landraces.

Recent genetic approaches for discovering trait-associated loci largely rely on high-quality reference genomes and high-density variant information (Thudi *et al.* 2021). Since the launch of the first human genome project (Lander *et al.* 2001), many genome assemblies have been published over the past two decades (Kersey 2019, Sohn and Nam 2018, Sun *et al.* 2022). Recent developments in DNA sequencing technology have further facilitated de novo genome assembly in non-model organisms and related species of model organisms (Li *et al.* 2017, Li and Harkess 2018). Currently, more than 300 rice varieties have been sequenced using the long-read sequencers PacBio Sequel/Sequel II and Nanopore MinION, and their genomes have been assembled (Choi *et al.* 2020, Du *et al.* 2017, Jain *et al.* 2019, Qin *et al.* 2021, Reuscher *et al.* 2018, Shang *et al.* 2022, Stein *et al.* 2018, Tanaka *et al.* 2020, Zhang *et al.* 2016, Zhou *et al.* 2020). Advances in sequencing technology have also accelerated whole-genome variant detection, enabling genome-wide association studies (GWAS) to discover causal loci/genes that explain phenotypic variations between germplasms (Xiao *et al.* 2017). Some national seed/gene banks have already begun to provide WGS data of publicly available genetic resources and have listed core collections (Milner *et al.* 2019, Tanaka *et al.* 2021). The core collection is a set of germplasms selected to efficiently evaluate the genetic diversity of an entire germplasm collection stocked in an organization by reducing the number of accessions to handle while maintaining genetic and phenotypic diversity as much as possible (Brown 1995). These minimized sets representing large, diverse collections allow us to explore large genetic variations with limited effort and facilitate the effective use of germplasms in seed/gene banks for breeding projects (Raturi *et al.* 2022).

Here, we report a genetic diversity analysis of rice varieties conserved in the Myanmar Seed Bank and a de novo genome assembly of the elite Myanmar *indica* variety, Inn Ma Yebaw (IMY). This study aimed to establish fundamental resources for facilitating molecular genetic studies to fully utilize Myanmar rice germplasms for rice breeding. For this purpose, we assembled a genetic diversity panel consisting of 250 *indica* accessions, designated as MIDP, standing for Myanmar *indica* diversity panel, from 610 Myanmar rice accessions based on high-resolution genotype data obtained by genotyping-by-sequencing (GBS). In addition, we assembled the de novo IMY genome sequence. Although our assembled genome has gaps, several quality assessments have indicated its contiguity and completeness in the gene-coding regions. Finally, GWAS of MIDP on apiculus pigmentation was performed to demonstrate the validity of MIDP using whole-genome variant information obtained by mapping WGS reads on the genome assemblies of IMY and the *japonica* variety Nipponbare (NB). Genome assembly and MIDP with variant information will facilitate deeper genetic research and breeding projects that utilize the untapped Myanmar rice germplasm.

## Materials and Methods

### Assembling the Myanmar indica diversity panel

The detailed process of assembling MIDP is described in the *Genotyping and assembling a diversity panel* section in Supplemental Text 2. In brief, 610 representative accessions were selected from the germplasms stocked in DAR, Yezin, Myanmar (Supplemental Table 1) and subjected to GBS for variant detection. We conducted a principal component analysis (PCA) using the obtained variant information (Supplemental Fig. 1) and selected 250 accessions as MIDP (Supplemental Table 2).

### Genome wide variant detection in MIDP

WGS of MIDP was performed by subjecting genomic DNA, which was extracted as described in the *Genotyping and assembling a diversity panel* section in Supplemental Text 2, to 150-bp paired-end sequencing using a DNBSEQ-500 sequencer in BGI Genomics, Shenzhen, China. The WGS generated sequencing read data of 5.15 Gb (34.4 M reads) per accession on average (Supplemental Table 2). Adapter- and quality-trimming of the sequencing reads were performed using SOAPnuke version 2.1.5 with the settings “-l 10 -q 0.1 -n 0.01 -f AAGTCGGAGGCCAAGCGGTCTTAGGAAGACAA -r AAGTCGGATCGTAGCCATGTCGTTCTGTGAGCCAAGGAGTTG” (Chen *et al.* 2018). The trimmed reads were mapped to the NB genome sequence (IRGSP-1.0, Kawahara *et al.* 2013) using the BWA-MEM algorithm with default settings (https://doi.org/10.48550/arXiv.1303.3997). Variant calling was conducted by following the best-practice workflow of germline short variant discovery introduced in the Genome Analysis Tool Kit version 4 (GATK4) webpage (https://gatk.broadinstitute.org/, https://doi.org/10.1101/201178). The resultant variants were filtered using the VariantFiltration module with the following criteria: QD < 2.0, QUAL < 30, SOR > 3.0, FS > 60.0, MQ < 40.0, MQRankSum < –12.5, and ReadPosRankSum < –8.0. Additionally, the arguments “--genotype-filter-expression ‘isHet == 1’ --genotype-filter-name ‘isHetFilter’” were also used for filtering. Only the biallelic SNPs that passed the filtering were extracted from the variant data using the SelectVariants module of GATK4 with the options “--select-type-to-include SNP --restrict-alleles-to BIALLELIC --exclude-filtered”. Furthermore, all heterozygous genotype calls were set to missing by setting “--set-filtered-gt-to-nocall” together with the options for the SelectVariants module. These processes retained 9,213,460 SNP markers in total.

### Population structure analysis

To determine the genetic relationships among the MIDP accessions, we conducted a PCA, population clustering analysis, and phylogenetic tree construction based on the obtained SNP data. The obtained SNP markers were filtered to retain only the markers showing more than 5% of the minor allele frequency and less than or equal to 10% of the missing rate. To reduce redundancy in variant information, the SNP markers were pruned by selecting one marker per 20-kb stretch. To confirm the classification of the MIDP accessions in the rice subpopulations, we also included the variant information obtained in the 3 K Rice Genome (3KRG) project (Wang *et al.* 2018). Representative accessions from the subpopulations including *indica*, aus/boro, basmati/sadri, *japonica*, tropical *japonica*, and temperate *japonica* were selected as listed in Supplemental Table 3. The variant information of the filtered MIDP and representative 3KRG datasets were intersected using Tassel 5.0 (Bradbury *et al.* 2007). We also inserted the variant information of the representative Myanmar rice varieties Paw San Hmwe (PSH) and Mote Soe Ma Kyway Kyay (MSMKK), which are a premium-quality aromatic variety and elite upland variety, respectively, into the intersected variant data. Finally, the intersected data contained 14,695 SNP markers. The resultant variant data were then converted to the R/genlight object using the vcfR2genlight() function in R/vcfR version 1.13.0 (Knaus and Grünwald 2017) and subjected to principal component analysis using the glPca() function of R/adegenet version 2.1.8 (Jombart and Ahmed 2011). The population structure analysis was conducted using the Bayesian clustering method implemented in *structure* program version 2.3.4 (Pritchard *et al.* 2000). The filtered MIDP variant data was converted to the specific format as input to structure as described in Supplemental Text 1. The reformatted variant data was subjected to the population structure analysis by setting the expected number of clusters to 1–10 using the “-K” parameter of *strucutre*. The neighbor-joining (NJ) tree was obtained using the nj() function in R/ape version 5.6.2 (Paradis and Schliep 2019). To reduce computational burden and redundancy in the variant data, we randomly selected 1422 SNPs from the filtered MIDP variant data. The distance matrix based on the Euclidean distance was calculated from the selected variant data and supplied to the nj() function as input. Branch partitioning on the tree was estimated by 1000-bootstrap replicates using the boot.phylo() function. An unrooted phylogenetic tree was visualized using Figtree software version 1.4.4 (http://tree.bio.ed.ac.uk/software/figtree/).

### De novo assembly of the IMY genome

DNA extraction and sequencing of IMY have been described in the *Sequencing the IMY genome* section in Supplemental Text 2. For the de novo assembly of the IMY genome, we constructed contigs from the obtained PacBio long reads using FALCON-unzip version 0.3.0 (Chin *et al.* 2016) with the settings described in the Supplemental Text 1. The constructed contigs were polished by two rounds of Pilon version 1.23 (Walker *et al.* 2014), with 332,005,644 short reads obtained via resequencing of the IMY genome using DNBSEQ-500 (see also the *Sequencing the IMY genome* section in Supplemental Text 2). The short reads underwent adapter- and quality-trimming, similar to the reads for MIDP. The alignment of the short reads to the contigs was achieved using BWA-MEM (https://doi.org/10.48550/arXiv.1303.3997) and supplied to Pilon as input for polishing. The polished contigs were anchored to 12 chromosomes using RaGOO version 1.1 (Alonge *et al.* 2019). Because RaGOO requires a reference genome for anchoring contigs, we conducted a phylogenetic analysis to find a genetically similar variety to IMY for which a high-quality genome assembly is available. The details of the phylogenetic analysis are described in the *Phylogenetic classification of IMY* section in Supplemental Text 2. In brief, IMY could be clustered with *indica* XI-3 varieties of the 3KRG accessions in a phylogenetic tree (Supplemental Figs. 2, 3). The local-ancestry inference also indicated that IMY was purely classified as XI-3 (Supplemental Table 4). Therefore, we selected the Tumba genome assembly, which is a near-gap-free genome assembly available for *indica* XI-3 varieties (Qin *et al.* 2021). The Tumba genome assembly was obtained from the Rice RC database (https://ricerc.sicau.edu.cn/) and supplied as a reference genome to RaGOO. Default settings were used for both Pilon and RaGOO.

### Creating gene models and identifying repeat elements

Gene models of the assembled IMY genome were created using the Comparative Annotation Toolkit (CAT) version 8.22 with the genome sequence and gene models of *Oryza sativa* ssp. *Japonica* cv. Nipponabre (NB) as reference data (Fiddes *et al.* 2018). The NB genome sequence in FASTA format and the latest gene models (Release 7) in GFF format were downloaded from the Rice Genome Annotation Project website (http://rice.uga.edu/) (Kawahara *et al.* 2013, Sakai *et al.* 2013). For ab initio gene modeling, RNA-seq reads of Tumba were aligned to the IMY genome using HISAT2 version 2.1.0 with flags “--dta” and an argument “-k 3” (Kim *et al.* 2019). The RNA-seq reads of Tumba in FASTQ format were obtained from the Genome Sequence Archive (https://ngdc.cncb.ac.cn/gsa/) by querying the project ID PRJCA000313 (Du *et al.* 2017). The obtained RNA-seq alignments (BAM files) and the NB gene models (a GFF file) were used as inputs in CAT. The following flags and arguments were specified “--augustus --augustus-cgp --augustus-species=rice”, while the default parameters were used for other options of CAT. Gene models were also generated using genBlast version 1.38 (She *et al.* 2009, 2011). A FASTA file of the protein sequences of the annotated NB genes was obtained from RAP-DB (https://rapdb.dna.affrc.go.jp). The FASTA file was supplied to genBlast as reference data with the following arguments and flags “-g T -r 1 -gff -cdna -pro”. Finally, we obtained the gene models, including those obtained using CAT and genBlast. Gene models generated by CAT and genBlast were merged as transcript variants of a gene if their coding sequences overlapped each other. The functions of the modeled genes were predicted using InterProScan version 5.57–90.0 (Jones *et al.* 2014, Paysan-Lafosse *et al.* 2023) and eggNOG-mapper v2 (version 2.1.9 and database version 5.0.2) (Cantalapiedra *et al.* 2021, Huerta-Cepas *et al.* 2019). The protein sequences of the modeled IMY genes were provided as inputs with the arguments and flags “-f tsv -goterms” and “--tax_scope Streptophyta” to InterProScan and eggNOG-mapper, respectively. For repeat elements, the assembled genome was analyzed using RepeatMasker version 4.1.0 with the RepBase RepeatMasker Edition (released on 01/27/2017) downloaded from www.girinst.org (Smit *et al.* 2013) and specifying “-excln -s -html -gff -no_is -species rice”. Putative centromeric regions were identified using a BLAST search using the rice satellite repeat sequence CentO (GenBank accession number: AF058902) as a query (Dong *et al.* 1998). The BLAST search was performed using the blastn function of BLAST+ version 2.9.0+ (Camacho *et al.* 2009) with default settings. The *Generating a circle plot* section in Supplemental Text 2 describes the procedure for generating a circle plot for the IMY genome assembly.

### Assembly quality assessment

The quality of the assembled IMY genome was assessed using the following tools. As a general evaluation, the contiguity of the assembly was measured using QUAST version 5.0.2 (Gurevich *et al.* 2013) with the flags “-e --large -k -f -s”. The completeness of the gene-coding regions was evaluated using BUSCO version 5.1.0, which searches for well-known single-copy conserved genes in a given assembly (Manni *et al.* 2021). We used default settings with the embryophyta database (Embryophyta_odb10) for the conserved gene search by BUSCO. The long terminal repeat (LTR) assembly index (LAI) was calculated to evaluate the completeness of repetitive regions in the assembly (Ou *et al.* 2018). For LAI calculation, LTR_retriever version 2.9.0 was used with the results obtained by applying LTRharvest version 1.6.1 and LTR_FINDER version 1.07 to the IMY genome assembly, as instructed in the GitHub repository (https://github.com/oushujun/LTR_retriever) (Ellinghaus *et al.* 2008, Ou and Jiang 2018, 2019). We also performed a reference-free quality assessment using Merqury version 1.3 (Rhie *et al.* 2020). The short reads from DNBSEQ-500 were supplied to Meryl version 1.3 to build a k-mer database and to measure k-mer occurrence in the reads (Miller *et al.* 2008). The resulting database was supplied to Merqury with a FASTA file of the IMY genome assembly. The optimal number for parameter k in Meryl was estimated using the best_k function, which was installed together with Meryl, by setting 400,000,000 as the genome_size argument, and k = 19 was chosen. All steps were performed according to the instructions available in the Merqury GitHub repository (https://github.com/marbl/merqury).

### Comparing assembly quality

The IMY genome assembly was compared with previously published high-quality genome assemblies of NB, Shuhui498 (R498), and Tumba. The genome FASTA files of Tumba and NB were obtained as described in previous sections: *De novo assembly of the IMY genome* and *Creating gene models and identifying repeat elements*, respectively. The FASTA file of the R498 genome assembly, a near-gap-free genome sequence of *indica* rice varieties (Du *et al.* 2017), was obtained from the Molecular Breeding Knowledge Base database (https://mbkbase.org/R498). Pair-wise genome collinearity was analyzed and visualized using D-GENIES version 1.2.0. The NB and IMY genome assemblies were supplied as query and subject genomes to D-GENIES with the default settings, respectively. To detect structural variations between genomes, we compared the IMY genome assembly with the three other assemblies using SyRI version 1.6 (Goel *et al.* 2019). Pair-wise whole genome alignment using minimap2 with the settings “-ax asm5 --eqx” was performed between the IMY assembly and each other assembly. The resultant alignment information was processed using SyRI with the default settings. The results were visualized using the genome comparison visualization tool plotsr version 0.5.4 (Goel and Schneeberger 2022).

To compare the quality of the assemblies, we also applied QUAST, BUSCO, RepeatMasker, and the LAI-calculation pipeline to the genome assemblies of NB, R498, and Tumba using the same settings and steps used for IMY.

### Calculation of linkage disequilibrium decay rate

To measure the linkage disequilibrium (LD) decay rate per chromosome, first, we calculated correlation coefficients (*r*) between the markers. To reduce the computational burden, 500,000 pairs of markers, each separated by a maximum of 10 Mb, were randomly selected from the obtained SNP markers. Pairwise *r* values were obtained using the LD() function of the R/genetics package (Warnes *et al.* 2021). The LD decay rate over physical distance was estimated by fitting the observed squared *r* values (*r*^2^) and marker distances (*d*) to an extension of the Sved (1971) model. The model formula is:

$$E\left[r^{2}\right]=\frac{r_{\text{high}}^{2}-r_{\text{low}}^{2}}{1+{dr}_{d}}+r_{\text{low}}^{2},$$

Where *r_d_*, $r_{\text{high}}^{2},$ and $r_{\text{low}}^{2}$ are the rate of decay, maximum observed *r*^2^, and minimum observed *r*^2^. The *r_d_*, $r_{\text{high}}^{2},$ and $r_{\text{low}}^{2}$ values for each chromosome were estimated by fitting the model to the observed *r*^2^ and *d* in each chromosome using the nls() function in the R/stats package (R Core Team 2023). In addition, the physical distance at which LD dropped to half (*d_LD_*_50_) was estimated using the following equation:

$$\frac{r_{\text{high}}^{2}+r_{\text{low}}^{2}}{2}=\frac{r_{\text{high}}^{2}-r_{\text{low}}^{2}}{1+d_{\text{LD}50}⸱r_{d}}+r_{\text{low}}^{2}$$

Solving this for *d_LD_*_50_ gives

$$d_{\text{LD}50}=\frac{1}{r_{d}}$$

The *d_LD_*_50_ value of each chromosome was calculated using the above equation with the estimated *r_d_*, $r_{\text{high}}^{2},$ and $r_{\text{low}}^{2}$ values for each chromosome. The genome-wide *d_LD_*_50_ was calculated by averaging the *d_LD_*_50_ values obtained for all chromosomes.

### Genome-wide association study

GWAS of apiculus pigmentation in MIDP was performed using the R package Gaston version 1.5.9 (Perdry and Dandine-Roulland 2022). Apiculus pigmentation was measured as a binary trait: colored or colorless. We conducted association tests on two variant datasets obtained by mapping reads onto the IMY and NB genome assemblies, respectively. Variant calling and primary filtering on SNP markers were conducted as described in the *Genome wide variant detection in MIDP* section. The obtained SNP markers were further filtered to retain only the markers showing more than 5% of the minor allele frequency and less than or equal to 20% of the missing rate. This filtering step retained 2,235,336 and 2,236,685 SNP markers for the variant datasets obtained using the IMY and NB genome assemblies, respectively. The retained SNP markers were subjected to the calculation of a genomic relationship matrix (GRM) using the GRM() function. Since the phenotype was a binary trait, we employed the logistic linear mixed model (LLMM) for association tests. Thus, each variant dataset and the obtained GRM were processed using the association.test() function with setting “method” and “response” to “lmm” and “binary”, respectively. No principal components were included in the model as fixed effects. The negative of the base 10 logarithm was calculated for each adjusted *p*-value, which is referred to as –log_10_(p). False discovery rates were also obtained using the p.adjust() function in R with the argument “method = ‘fdr’”. A significant quantitative trait locus (QTL) was defined as a genomic region that contained at least one marker exceeding the significant threshold and multiple markers with scores higher than the suggestive threshold, all falling within a range of the genome-wide *d_LD_*_50_ from a peak association marker. LD heatmaps were constructed using the R/LD heatmap package (Shin *et al.* 2006) to visualize local LD blocks at the significant QTLs.

### Data availability

A genome browser and BLAST search service for the IMY genome assembly are available in the IMY genome database at http://rginfres.rib.okayama-u.ac.jp. The IMY genome sequence and all read data, including long reads by PacBio and short reads by BGISEQ-500, were deposited with links to BioProject accession number PRJDB15417. The IMY genome sequence in FASTA format, annotation information in GFF format, and variant information of MIDP in VCF format can be found in the IMY genome database. The Supplemental Text 1 contains the codes used in this study.

## Results

### Assembling the diversity panel MIDP

To organize a diversity panel from diverse rice germplasms in Myanmar for enabling genetic survey by GWAS, 610 representative accessions were initially selected from a rice germplasm collection stocked in the Seed Bank of DAR (Supplemental Table 1). The representative accessions were selected based on their phenotypic variation and geographical origins, including representative Myanmar rice varieties: IMY, an elite lowland variety; MSMKK, an elite upland variety; and PSH, a premium aromatic variety. To evaluate the population structure of the 610 accessions, we conducted a PCA and visualized principal component scores based on the genotypes obtained by GBS (Supplemental Fig. 1). We could find roughly three clusters each of which included IMY, MSMKK, and PSH, respectively. The majority of the accessions were clustered together with IMY. These subpopulation clusters potentially lead to the detection of false positive associations in GWAS. Therefore, we chose the accessions clustered around IMY as a diversity panel because the subset could let us avoid stratification in the population while keeping a population size. Out of the 610 accessions, 250 accessions were selected as members of the diversity panel MIDP (Supplemental Table 2).

### Population structure of the diversity panel

**Fig. 1.**
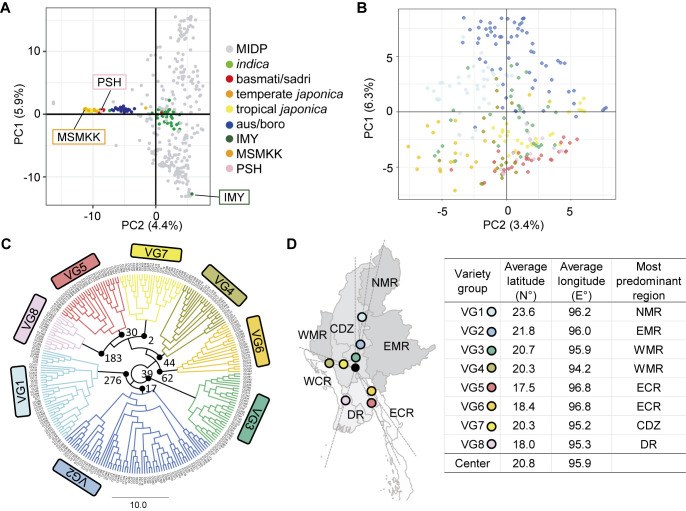
Characterization of MIDP based on the variant information. (A) The PCA plot of the PC1 and PC2 scores for the MIDP accessions and reference 3KRG accessions belonging to the known subpopulations, *japonica*, *indica*, aus/boro, and aroma (basmati/sadri). The representative elite Myanmar varieties (PSH, IMY, and MSMKK) were also included. (B) The PCA plot of the PC1 and PC2 scores only for MIDP. The dot colors correspond to the color code of VGs shown in panel C. The percentages in the parentheses indicate the contribution ratios of principal components. (C) The NJ tree of MIDP. The clades in different colors represent the classification of the accessions into the eight VGs (VG1-8). The digits near the black dots represent the number of iterations supporting the branchings at 1000-bootstrap replicates. (D) The colored dots on the Myanmar land map show the mean latitudes and longitudes of the collection places of the VG varieties. The colored dots spreading radially along the dotted lines from the overall mean of the latitudes and longitudes of MIDP (black dot) suggests that the grouping of the VGs reflects the phylogeographical relationships of Myanmar rice varieties.

To characterize MIDP, the population structure and phylogenetic relationship were visualized using genome-wide variant data obtained by mapping WGS of MIDP of the 250 accessions on the NB genome. First, we performed a PCA on the variant data of 14,695 SNPs obtained by intersecting the variant data of MIDP and 3KRG to classify the accessions into the known population structure observed in the cultivated rice (Fig. 1A). The first principal component (PC1) represented the diversity in MIDP. Since the variant data of MIDP only contained variant sites observed within MIDP, the intersection of the variant data might preferentially retain the variants representing the diversity in MIDP, whereas the variant sites observed only in the 3KRG dataset used in this study could be filtered out. As a result, PC1 represented the diversity in MIDP in the PCA, but not the diversity between subpopulations, which is typically expected to be reflected in PC1. Nonetheless, the PC2 axis showed an apparent separation between the *indica*, aus, and *japonica* varieties of 3KRG. In contrast, the MIDP accessions were predominantly clustered around the *indica* 3KRG accessions. These results suggest that the MIDP accessions selected based on their genetic similarity to IMY belong to the *indica* subpopulation of rice.

To confirm the population structure within MIDP, we performed PCA only using the MIDP variant data (Fig. 1B). The plot on the PC1-PC2 plane showed no apparent clusters. In addition, PC1 and PC2 accounted for only 6.3% and 3.4% of the total variance, respectively (Supplemental Fig. 4). The PC1 and PC2 scores showed weak correlations with the latitude and longitude of the geographical origin of the accessions (*r*_pc1-latitude_ = 0.39 and *r*_pc2-longitude_ = 0.22). Environmental differences along the latitudes might primarily cause genetic variations in Myanmar rice landraces but not genetically separate the accessions. The population structure analysis also suggested no apparent subpopulation structure in MIDP (Supplemental Fig. 5). These results suggest that the genomes of MIDP were well shuffled under the Hardy–Weinberg equilibrium (HWE) and also indicate that MIDP has an ideal genetic structure for GWAS theoretically assuming the majority of variants follow HWE.

Breaking down a core collection into lower hierarchical levels helps us explore the genetic diversity of the collection. For example, we can choose a representative accession from each subgroup of the core collection and develop biparental cross populations for QTL identification and breeding. Therefore, we classified the accessions into eight varietal groups (VG1-8) based on the NJ tree, although the clustering was limitedly supported by the bootstrap analysis (Fig. 1C, Supplemental Table 2). The average latitude and longitude of each VG were calculated based on the available records of the geographical origins of the accessions (Supplemental Table 2) and plotted on the Myanmar land map (Fig. 1D). As indicated by the correlation between the geographical origins and the PC scores of the accessions, the VGs classified by the NJ tree showed geographical separation, especially along the latitude (Fig. 1D). To link the VGs and agroecological environments in Myanmar, we tested which VG predominantly included accessions cultivated in different geographical regions: the delta region (DR), central dry zone (CDZ), eastern mountainous region (EMR), northern mountainous region (NMR), western mountainous region (WMR), eastern coastal region (ECR), and western coastal region (WCR) (Fig. 1D). We determined, for each VG, the region in which the accessions were predominantly cultivated based on the observed and expected number of accessions sampled from each geographical region (Supplemental Table 5). The region that showed the largest observed/expected ratio was assigned to each VG. We found that VG1-8 predominantly contains accessions cultivated in NMR, EMR, WMR, WMR, ECR, ECR, CDZ, and DR, respectively. The largest number of accessions (66) was classified as VG2 and was predominantly cultivated in EMR. In addition, the majority of the accessions sampled from EMR (63%) were classified as VG2 (Supplemental Table 5). These results indicate that the accessions genetically fitting the cultivation in EMR are preferentially cultivated in EMR as expected. Similar trends were observed between VG1-NMR, VG3-WMR, VG4-WMR, VG5-ECR, and VG6-ECR. Although these VGs include only less than half of the accessions cultivated in the corresponding regions, the accessions sampled in mountainous and coastal regions were preferentially classified in VGs that were predominantly cultivated in mountainous and coastal regions, respectively. These results might support our finding of the correlation between the genetic variations of MIDP and the latitudes of their origins, which roughly align with the mountainous-coastal axis. In contrast, DR was assigned to VG8 that had only 14% of the accessions originally sampled from the DR (Supplemental Table 5). This result implies that the accessions cultivated in the DR have been largely adopted from accessions originally established in other regions. A similar result was observed for the relationship between VG7 and CDZ, where the accessions sampled in CDZ were also well classified in the other VGs (Supplemental Table 5).

### Overview of the IMY genome assembly

**Fig. 2.**
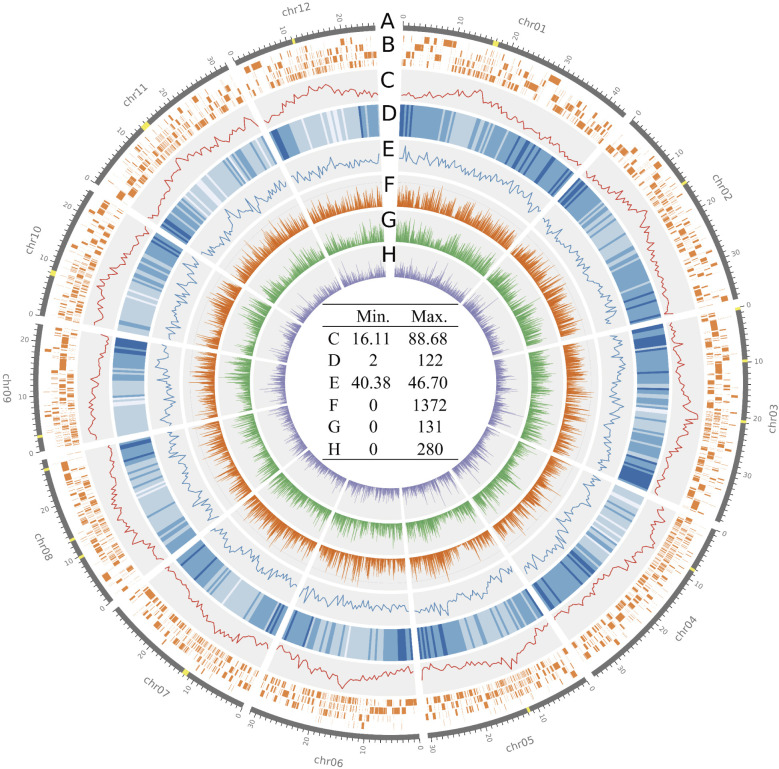
Circle plot of the IMY genome assembly. The circle plot shows contig tiling in the assembly, CentO sequence positions, and genome statistics. (A) The 500-kb bins containing the CentO sequence are highlighted in yellow on the outermost circle of the plot. (B) The second circle indicates the positions and lengths of the assembled contigs. (C–E) The next three circles represent the density of repeat elements, number of genes, and GC content in the 500-kb bins. (F–H) The last three circles are bar plots indicating the number of SNPs, insertions, and deletions identified by SyRI in the 100-kb bins of the assembly. The maximum and minimum values in each circle are listed at the center of the plot.

IMY was chosen as the representative accession for MIDP because it is an elite cultivar in Myanmar and the members of MIDP were selected based on genetic similarity to IMY. Thus, it was ideal to obtain a high-quality genome assembly of IMY to establish a basis for molecular genetic research for rice breeding utilizing Myanmar germplasms. As a consequence of anchoring contigs assembled from 1,202,170 long reads generated by the PacBio Sequel (Supplemental Table 6), we obtained an IMY genome assembly consisting of 2888 contigs anchored into the 12 chromosomes, reaching 380,400,034 bp in total (Fig. 2, Table 1). The CentO sequence, which is known to repeat in tandem in the centromeric regions of chromosomes, was found in all chromosomes except for chromosome 6 (Fig. 2A). Relatively short contigs were frequently observed around the centromeric regions (Fig. 2B). These centromeric regions harbored more repeat sequences than the distal ends of the chromosomes (Fig. 2C). A total of 41,546 genes were annotated in the genome. We found that the genome has genes at a higher density at the distal ends than those in the centromeric regions (Fig. 2D). The overall GC content was 43.34%, although the local GC content ranged from 40.47% to 46.33% (Fig. 2E). The genome sequence comparison between IMY and NB revealed SNPs, insertions, and deletions throughout chromosomes, which showed similar distributions and relatively low frequencies around the centromeric regions (Fig. 2F–2H). A reference-free k-mer-based assembly quality assessment was performed using Merqury. The resultant k-mer spectra indicated that the majority of k-mers were observed at one location in the assembly, whereas read-only k-mers were mainly found at low frequencies (Supplemental Fig. 6). A single peak formed by the once-observed k-mers in the spectra indicated a high homozygosity of the genome. In addition, our IMY genome assembly scored a Merqury quality value (QV) of 31.42 and a Merqury k-mer completeness of 96.89. Since a recent publication reported that an improved genome assembly of the *indica* variety 93-11, which is a well-known high-quality genome assembly, scored a QV of 31.55 (Wang *et al.* 2022), our assembly was of sufficient quality as a high-quality genome assembly.

### Comparing the genome assembly qualities

We compared the IMY genome assembly with previously published high-quality genome assemblies, including the assemblies of NB and R498, in addition to Tumba (Supplemental Table 7). Compared to our IMY genome assembly, the assemblies of NB, R498, and Tumba consisted of a small number of contigs: 243, 40, and 55, respectively (Table 1). As expected, the largest contig length and N75 also indicated less contiguity of the IMY genome than the others. In contrast, the BUSCO score (single + duplicated) of the IMY assembly was 98.27, which was higher than that of the R498 assembly (Table 2). Even though NB and Tumba had slightly higher BUSCO scores (98.39 and 98.58) than IMY, the BUSCO analyses indicated the high completeness of gene-coding regions in our assembly. In contrast to the BUSCO analyses, repeat element analyses revealed differences between the IMY assembly and the others (Supplemental Table 8). Repeat elements were found in 45.91% of the IMY assembly, whereas the NB, R498, and Tumba assemblies contained 47.76, 49.66, and 49.94%, respectively (Supplemental Table 8). In particular, the proportion of retroelements and LTRs in the IMY assembly was smaller than that in the other assemblies, whereas the IMY assembly showed a similar or even higher proportion of DNA transposons than the others. In addition, the IMY assembly had the lowest LAI of the four assemblies tested (Supplemental Table 8). Retroelements and LTRs are transposable elements that copy themselves to other locations, whereas DNA transposons move from one location to another (Bourque *et al.* 2018). Therefore, high-similarity sequences could be present at multiple locations in a genome because of the activity of retroelements and LTRs. These repetitive sequences prevent us from assembling contiguous contigs if the obtained reads are not long enough. These results indicate that the IMY assembly lacks some sequences of repeat elements, especially retrotransposons, whereas the gene-coding regions and non-repetitive regions have been assembled at high quality.

### Characterizing the genome structure of IMY via structural variant analyses

We compared structural variations among the four genome assemblies. First, we visualized the collinearity between the IMY and NB assemblies. As previously reported (Du *et al.* 2017, Stein *et al.* 2018), a large inversion was found on chromosome 6, whereas the IMY and NB assemblies showed high collinearity on the other chromosomes, even without large inter-chromosomal rearrangements (Supplemental Fig. 7). To further evaluate structural variations, genome assemblies were compared using SyRI (Goel *et al.* 2019). In addition to the inversion in chromosome 6, we found many small inversions, translocations, and duplications, as well as simple insertions and deletions between IMY and NB (Supplemental Fig. 8, Supplemental Table 9). Approximately 77.7% of the IMY assembly was classified as syntenic regions with the NB assembly, whereas 18.35% of the IMY genome sequence was not aligned with the NB assembly, indicating high divergence in these sequences (Supplemental Table 9). As expected from the fact that only NB is a *japonica* variety and the others are *indica* varieties, R498 and Tumba showed fewer variations in comparisons to IMY than NB did (Supplemental Fig. 9, Supplemental Table 9). Because the Tumba assembly was used as a reference for scaffolding, the fewest variations were observed between IMY and Tumba (Supplemental Table 9). Relatively large inversions were found between IMY and R498 on chromosomes 6, 8, and 11, which were not found in the comparison between IMY and Tumba (Supplemental Fig. 9). In addition, IMY-specific inversions and duplications were detected on chromosomes 2, 5, and 10 (Supplemental Fig. 9). The IMY assembly was syntenic with the R498 and Tumba assemblies in 89.07 (339.4 Mb) and 91.52% (348 Mb) of the sequences, respectively, whereas 8.48 (32.3 Mb) and 7.21% (27.5 Mb) of the IMY genome sequence were not aligned to the R498 and Tumba genome sequences, respectively (Supplemental Table 9). In addition to these structural variations, we detected SNPs, insertions, and deletions at approximately 600 kb, 2 Mb, and 2.8 Mb of nucleotides in the IMY genome compared to either R498 or Tumba (Supplemental Table 9). Hence, although Tumba is genetically close to IMY, these genome assemblies showed a non-negligible proportion of genome-scale structural variations as well as local variations, including SNPs, insertions, and deletions.

### Demonstration of GWAS in MIDP

**Fig. 3.**
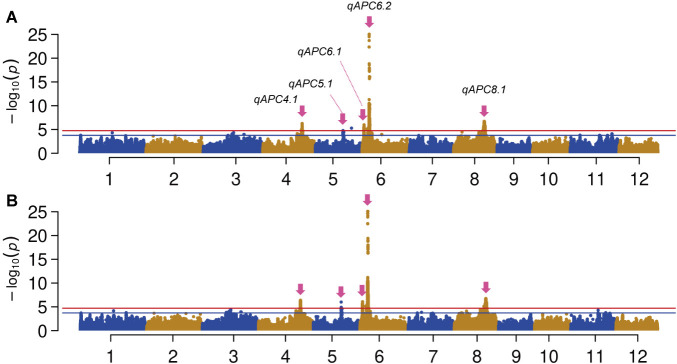
Manhattan plots of GWAS on apiculus pigmentation using the IMY and NB genomes. Manhattan plots for the GWAS using the IMY (A) and NB genomes (B) as a reference, respectively. The red and blue horizontal lines represent suggestive and significant threshold levels at FDR = 0.05 and 0.01, respectively. Significant QTL locations are represented by magenta arrows with labels indicating the QTL names.

Finally, the performance of MIDP for GWAS was evaluated for apiculus color (APC), which is a measure of anthocyanin accumulation at the distal ends of rice spikelets. We detected 7855 significant associations in total across the 12 chromosomes in the GWAS using the IMY genome (Fig. 3A). As the genome-wide *d_LD_*_50_ was 96,521 bp (Supplemental Table 10, Supplemental Fig. 10), we set the range of the stretch to define a significant QTL 96,521-bp upstream and downstream of a peak association marker. Based on these criteria, five significant QTLs were obtained (Fig. 3A, Table 3). The significant and suggestive association markers near the peak association markers in the five QTLs were verified to be in linkage disequilibrium (Supplemental Figs. 11–15). Clear LD block structures were observed at the QTLs except for *qAPC8.1*. To validate the IMY genome as a resource for GWAS and confirm the reliability of the GWAS result, we also conducted a GWAS using the NB genome as a reference for variant calling. The GWAS using the NB genome resulted in highly similar association signals throughout the chromosomes compared to those observed in the GWAS using the IMY genome (Fig. 3B). These results demonstrate that the IMY genome assembly has enough quality and contiguity comparable to the NB genome assembly as a reference genome for GWAS.

Next, we measured correlations between the genotypes at the peak association markers detected in the GWAS using the IMY genome. Mild but statistically significant correlations (*p* < 0.001) were observed between *qAPC6.2* and the other QTLs (Table 3). In addition, the association tests were performed using the regression model including the genotype at *qAPC6.2* as a fixed effect. We employed the general linear mixed model (GLMM) for these tests because the LLMM with the fixed effect resulted in severe *p*-value inflations across the entire genome (Supplemental Fig. 16A). Similar to the association tests using the LLMM without the fixed effect (Fig. 3A), the five significant QTLs were observed even using the GLMM without the fixed effect (Supplemental Fig. 16B). The association tests using the GLMM with the fixed effect resulted in the elimination of significant association signals at the five QTLs (Supplemental Fig. 16C). These results suggested that the strong signal at *qAPC6.2* and its genotypic correlations with the other QTLs led false positive associations. Therefore, only *qAPC6.2* was considered the QTL showing the true positive association with the presence or absence of apiculus pigmentation in MIDP. Following this, we examined the gene annotations within the LD block of *qAPC6.2* in the IMY genome assembly and identified the IMY ortholog of *OsC1* close to the peak association marker (Supplemental Fig. 14). A previous study revealed that *OsC1* is an R2R3-Myb transcription factor (TF) that regulates anthocyanin synthesis, and a 10-bp deletion in the coding sequence resulting in a frameshift causes colorless apiculi (Saitoh *et al.* 2004). Although the peak association was not located on the *OsC1* gene coding region, the 10-bp deletion was found in the variant data of MIDP (Supplemental Fig. 17). In addition, the genotype at the peak association marker of *qAPC6.2* showed a perfect correlation with the presence or absence of the 10-bp deletion at least for the accessions having non-missing genotype calls at both variant sites (Supplemental Table 11). Out of 161 accessions having reference homozygous genotypes at *qAPC6.2* indicating the presence of the 10-bp deletion, 159 accessions showed colorless apiculi (Supplemental Fig. 18). Although the alternative homozygous genotypes at *qAPC6.2* indicated the presence of the functional *OsC1* gene, four out of 61 accessions showed colorless apiculi (Supplemental Fig. 18). This result implies that *OsC1* predominantly explains the phenotypic variation of apiculus coloration in MIDP, whereas other gene(s) also affects apiculus pigmentation in this population. Indeed, the association tests using the regression model including the genotype at *qAPC6.2* as a fixed effect showed significant associations at two loci: *qAPC8.2* and *qAPC12.1* (Table 3, Supplemental Fig. 16). Out of five significantly associated markers, four were found at *qAPC12.1* whereas only one located at *qAPC8.2*. These QTLs had low correlations with *qAPC6.2* (Table 3, Supplemental Table 11). Interestingly, we found that the four accessions that showed colorless apiculi under the presence of the functional *OsC1* had alternative homozygous genotypes at both loci (Supplemental Fig. 18, Supplemental Table 11).

## Discussion

### Quality of the genome assembly

WGS reads obtained using third-generation sequencers such as PacBio Sequel/Sequel II and Nanopore MinION (Jiao and Schneeberger 2017) are now fundamental data for de novo genome assembly. In addition, recent genome assembly projects often use additional data that provide information about the long-distance structural relationships of genome sequences, such as Hi-C, optical mapping (Lam *et al.* 2012, van Berkum *et al.* 2010) as well as linked-reads by 10x Genomics (Weisenfeld *et al.* 2017). In contrast, we reported an IMY genome assembly constructed only using long-read sequence data obtained using PacBio Sequel. Although short reads were also obtained for IMY, these data were only used for error correction of the assembled contigs. The total amount of long-read data was approximately 30.4× coverage, less than the recommended amount for de novo assembly, which is 40–50× (Li and Harkess 2018). Our assembled genome contained 2876 gaps between 2888 anchored contigs (Table 1). Nevertheless, assembly quality assessments revealed that the gene-coding regions of our assembly were nearly complete, whereas there were many missing sequences in the repeat elements (Table 2, Supplemental Table 8). In many breeding projects, researchers and breeders have primarily focused on the gene-coding regions. Usually, highly repetitive sequences are not the primary targets for genomics-assisted breeding (Varshney *et al.* 2021). Hence, our genome assembly provides high-quality genomic information for breeding and genetic studies.

### The potential of MIDP and the IMY genome

Our GWAS results illustrate the potential of MIDP and the IMY genome assembly as genetic and genomic resources for genetic researches. In our GWAS of apiculus pigmentation in MIDP, we successfully identified the promising candidate gene *OsC1* at *qACP6.2*. The metabolic and genetic pathways that regulate anthocyanin biosynthesis in plants have been well-described (Holton and Cornish 1995, Xia *et al.* 2021). The anthocyanin pigmentation in rice has been reported to be regulated by three types of regulatory genes: R2R3-MYB TF, bHLH TF, and WD40-repeat TF (Xia *et al.* 2021). *OsC1* encoding an R2R3-MYB TF is a determinant factor of anthocyanin pigmentation in various organs, including the apiculus (Zheng *et al.* 2019). In MIDP, all accessions except for two accessions harboring the alternative allele at the peak association marker in *qAPC6*.*2* showed colorless apiculi. We also found that the genotypes at the peak association marker perfectly correlate with the causal 10-bp deletion in the *OsC1* gene, leading to a lack of apiculus coloration (Supplemental Table 2). This suggests that *OsC1* is the primary factor responsible for the variation in apiculus pigmentation within this population. In addition to *OsC1*, we could identify two QTLs (*qAPC8.2* and *qAPC12.1*) potentially controlling apiculus pigmentation in a complemental manner (Supplemental Figs. 16, 18). However, *qAPC8.2* was supported by only one SNP marker that exceeded the significant threshold, whereas *qAPC12.1* had three significantly associated markers nearby the peak association marker. In addition. we employed the GLMM for the binary trait, which is an inappropriate application. Therefore, although these QTLs successfully explained the colorless apiculi in the genetic background of the functional *OsC1*, further careful validation to confirm the possibility to be false positives and an additional genetic analysis are required. Nevertheless, these results demonstrate that MIDP and its variant data have sufficient genetic variation and data quality to conduct genetic surveys of Myanmar *indica* varieties. In addition, the similarity in association signals in the GWAS results using the NB and IMY genome indicates that the quality of our IMY genome assembly is sufficient for GWAS (Fig. 3). Although various genomic assemblies of rice landraces have been released (Choi *et al.* 2020, Qin *et al.* 2021, Shang *et al.* 2022, Stein *et al.* 2018, Zhou *et al.* 2020), the IMY genome assembly is the first genome assembly of a Myanmar landrace. The IMY genome assembly would not only be counted as a new collection of rice genome assemblies but could also be a fundamental resource for genetic research on untapped landraces. Overall, the genetic and genomic resources developed in our study will accelerate molecular genetic research exploring the gene pool of Myanmar landraces for rice breeding worldwide.

## Author Contribution Statement

AY and MA conceptualized and administrated the study. TF performed GBS on Myanmar rice accessions, IMY genome assembly, annotation, and quality assessment. MMH, TF, and YY performed population structure analyses and GWAS. YY and MA performed DNA extraction and WGS of MIDP. OMS and MST administrated the Myanmar activity of this study. Plant materials were prepared by KTW, OMS, MMH, ALLH, HY, SM, and YY. The apiculus color evaluation was performed by KTW, OMS, and SM. The manuscript was originally written by TF and YY and reviewed by YY, HY, and AY.

## Supplementary Material

Supplemental Figures

Supplemental Tables

Supplemental Text

## Figures and Tables

**Table 1.** Statistical summary of the genome assemblies

	IMY	NB	R498	Tumba
# contigs	2888	243	40	55
Largest contig	2,262,336	17,510,758	30,029,857	30,153,519
Total length	380,764,276	373,129,634	391,620,437	391,765,502
GC (%)	43.24	43.56	43.61	43.64
N50	364,379	7,711,345	15,378,818	12,840,232
N75	159,796	3,630,979	9,965,796	8,857,783
L50	279	17	9	12
L75	663	34	17	21

**Table 2.** BUSCO scores

Database		IMY	NB	R498	Tumba
Embryophyta_odb10 (1614 BUSCOs)	Single-copy	94.30	96.47	94.92	96.16
	Duplicated	3.97	2.11	2.35	2.23
	Fragmented	0.93	0.87	1.80	1.05
	Missing	0.81	0.56	0.93	0.56

**Table 3.** Summary of the QTLs for apiculus pigmentation

QTL name	Chr.	Position*^a^*	Allele*^b^*	–log10(p)	Correlation*^c^*
*qAPC4.1*	4	26,548,658	G/A	6.26	0.462
*qAPC5.1*	5	18,665,049	C/T	4.77	0.397
*qAPC6.1*	6	1,380,945	A/T	5.98	0.469
*qAPC6.2*	6	4,866,454	T/C	25.05	1.000
*qAPC8.1*	8	20,763,013	G/A	6.71	0.504
*qAPC8.2^d^*	8	7,780,702	A/T	8.05	0.141
*qAPC12.1^d^*	12	958,898	A/G	9.37	0.134

*^a^* Physical positions in the IMY genome assembly. *^b^* Reference/alternative alleles. Underlined letters indicate alleles confer apiculus coloration. *^c^* Correlation coefficients were measured between the genotypes at the peak association markers of *qAPC6.2* and each of the QTLs. *^d^* These QTLs were detected in the GWAS including the genotypes at *qAPC6.2* as a fixed effect.
