# Supplementary material for: Development of genomic and genetic resources facilitating molecular genetic studies on untapped Myanmar rice germplasms
Source: Breed Sci. 2024 Mar 22;74(2):124–37. doi: 10.1270/jsbbs.23077 (PMC11442107; doi:10.1270/jsbbs.23077)
Supplement: Supplementary file 1 — Supplemental Figures [file 74_124_s1.pdf]

doi: 10.1270/jsbbs.23077

Supplemental Figures

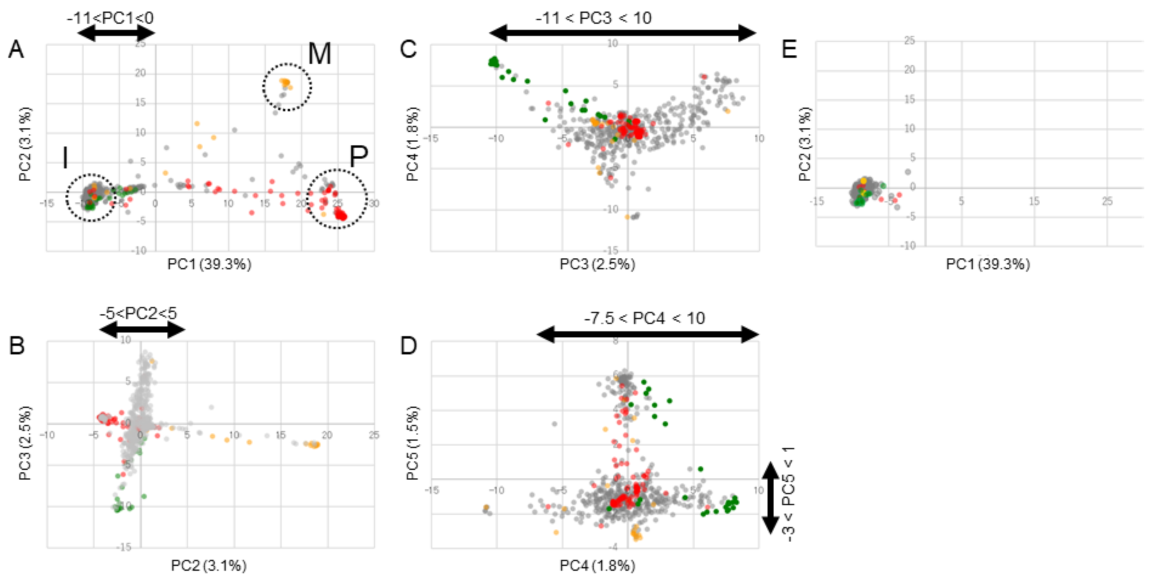

Supplemental Fig. 1. Principal component analysis of the 610 Myanmar accessions. (A-D) The PCA plots show the population structure of the 610 Myanmar accessions on the planes of the combinations of principal components (PC1-5). The clusters on the PC1-PC2 plane are indicated by dotted circles labeled as I, M, and P representing the clusters including IMY, MSMKK, and PSH, respectively. The black double arrows indicate the ranges of PC scores for screening a diversity panel. (E) The PCA plot shows the clustering of the screened 250 accessions on the PC1-PC2 plane with the same scale as depicted in panel A. The red, green, and yellow dots represent the accessions related to IMY, MSMKK, and PSH, whereas the other accessions were represented by gray dots. The percentages in the parentheses indicate the contribution ratios of principal components.

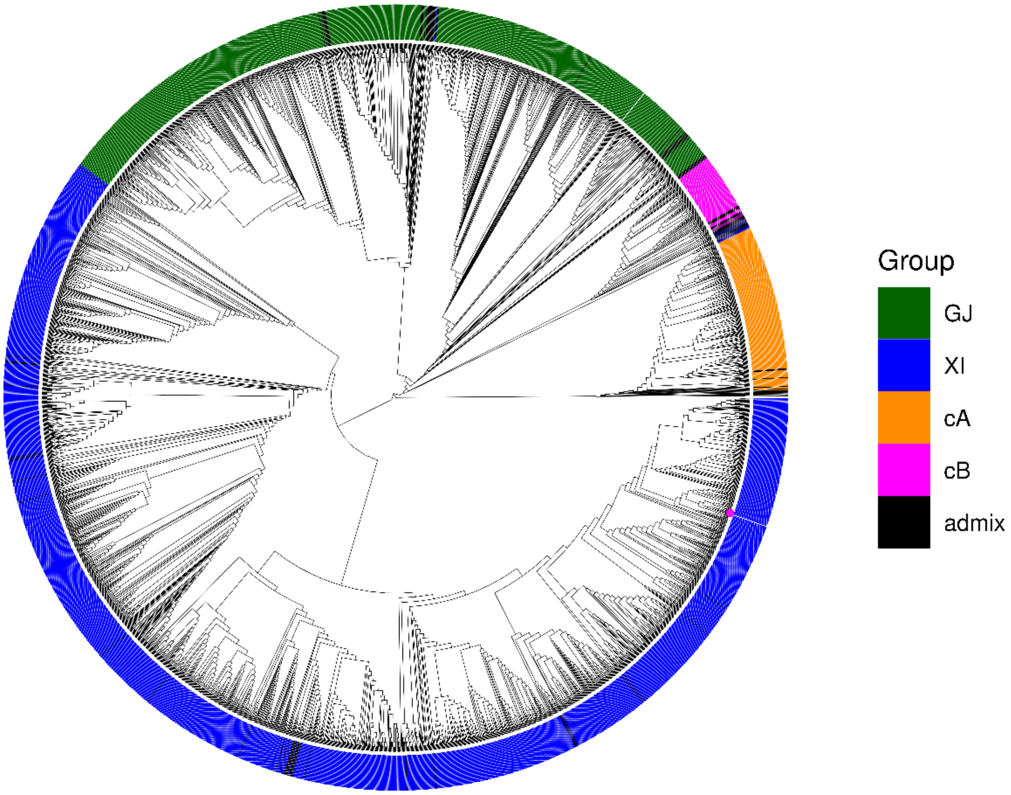

Supplemental Fig. 2. Phylogenetic tree of 3KRG with IMY.

The phylogenetic tree was created based on the filtered whole genome variant information including 3KRG and IMY. The filled magenta circle indicates the position of IMY in the tree. Although 3KRG was classified into nine subgroups, we classified them into 5 groups in this plot. GJ includes GJ-trp, GJ-sbtrp, GJ-tmp, and GJ-admix, while XI contains XI-1A, XI-1B, XI-2, XI-3, and XI-admix.

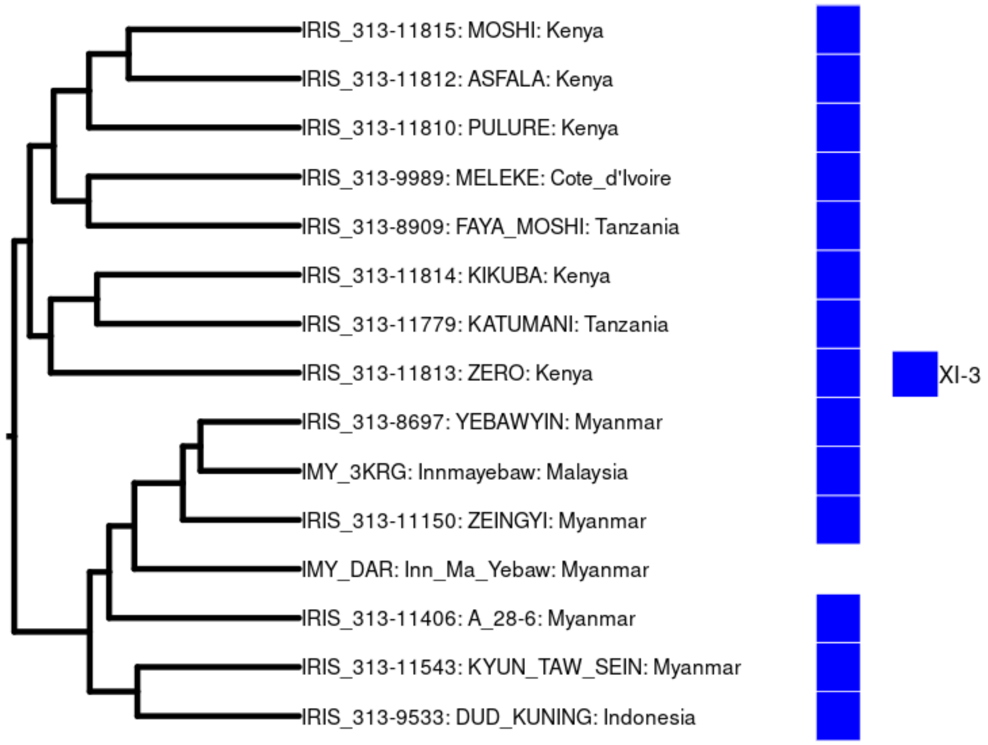

Supplemental Fig. 3. Subset of the phylogenetic tree of 3K-RG with IMY.

The phylogenetic tree was created based on the filtered whole genome variant information including 3KRG and IMY. The tree only around IMY was shown here. The tip labels of the tree consist of unique IDs in the dataset, variety names, and counties of origin, which are separated by colons. IMY_DAR indicates the IMY sequenced in this study, while IMY_3KRG is an IMY that was sequenced in the 3KRG project. Interestingly, IMY_3KRG was listed as a Malaysian variety although it was closely grouped with IMY_DAR. This clade only contains accessions classified in XI-3.

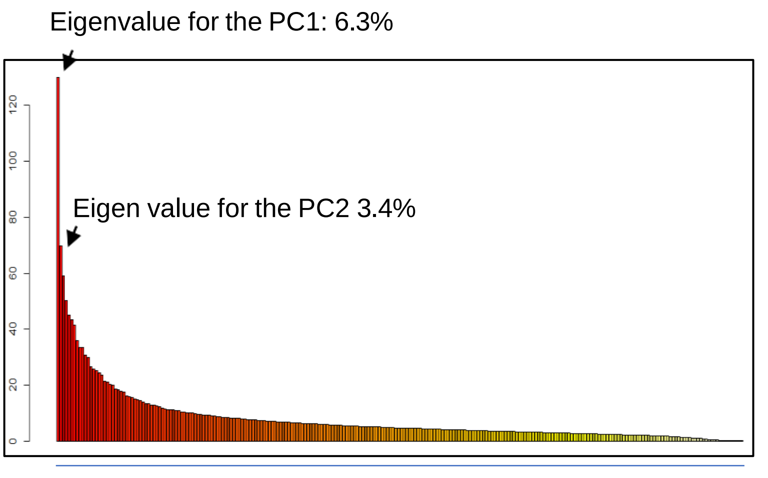

Supplemental Fig. 4. Distribution of eigenvalues in PCA using genotypic data at 14,695 loci.

The highest 256th of eigenvalues were shown in this figure.

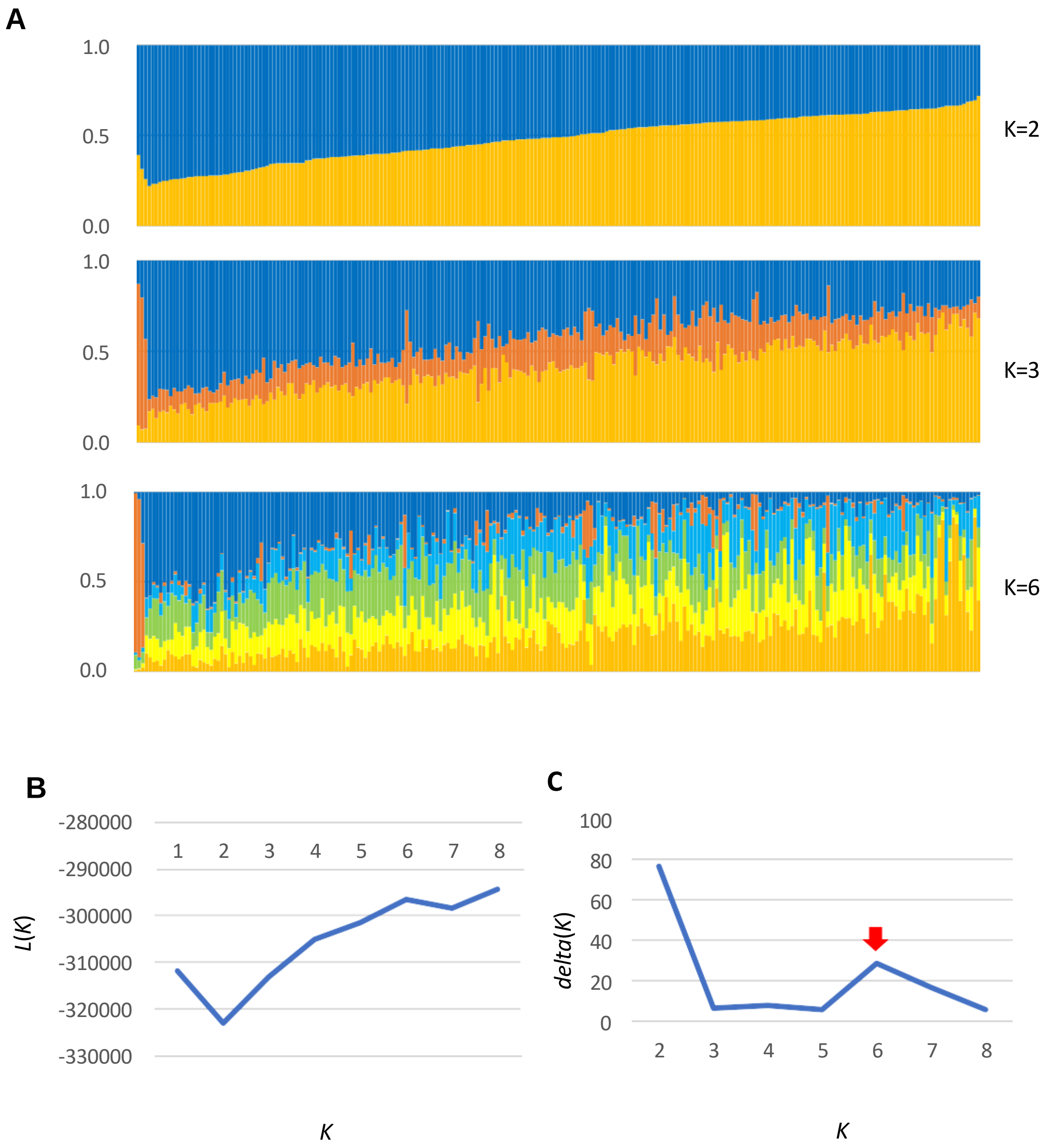

Supplemental Fig. 5. No apparent subpopulation structure in the Bayesian clustering of MIDP.

(**A**) Barplots represented in the subpopulation portions of the MIDP accessions when the number of subpopulations (*K*) is assumed as *K*=2, 3, and 6. (**B**) Average likelihood at each of *K*. The average likelihood at K=2 showed a minimum, demonstrating that *K*=2 is not an appropriate estimation. (**C**) Δ*K* analysis for evaluation of subpopulation differentiation. In general, Δ*K* statistic at *K*=2 show the local maximum. Although the local maximum of the Δ*K* statistic appeared at *K*=6, the Δ*K* statistic at *K*=6 is lower than that at *K*=2, demonstrating that the subpopulation differentiation at *K*=6 is not sufficiently supported.

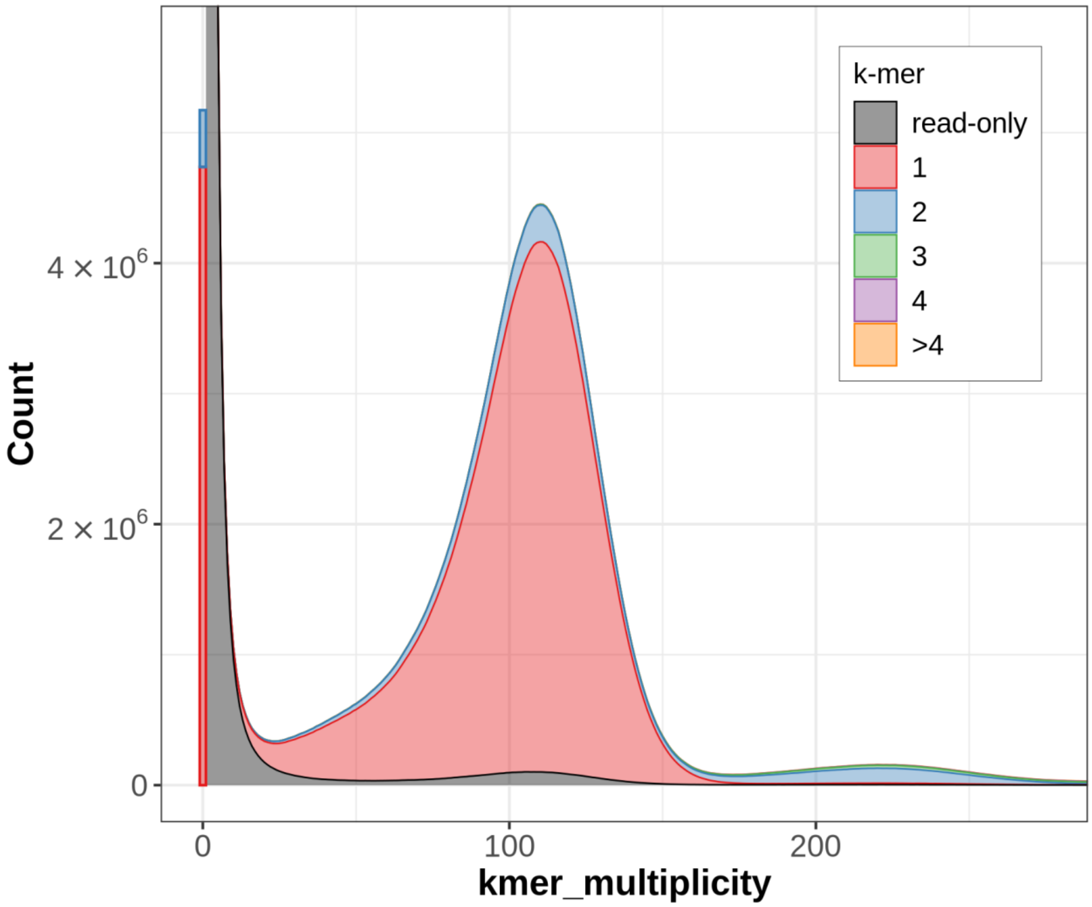

Supplemental Fig. 6. K-mer spectra of the IMY genome assembly.

The density plot indicates the density (counts) of the k-mers that were observed in the short-read data at the frequencies indicated on the x-axis. The different colors represent the difference in the number of k-mers in the IMY genome assembly. Since read-only k-mers (shown as a gray area) were mainly found at less than 25 multiplicity, the k-mers with kmer_multiplicities less than 25 in the plot were considered the results of sequencing errors.

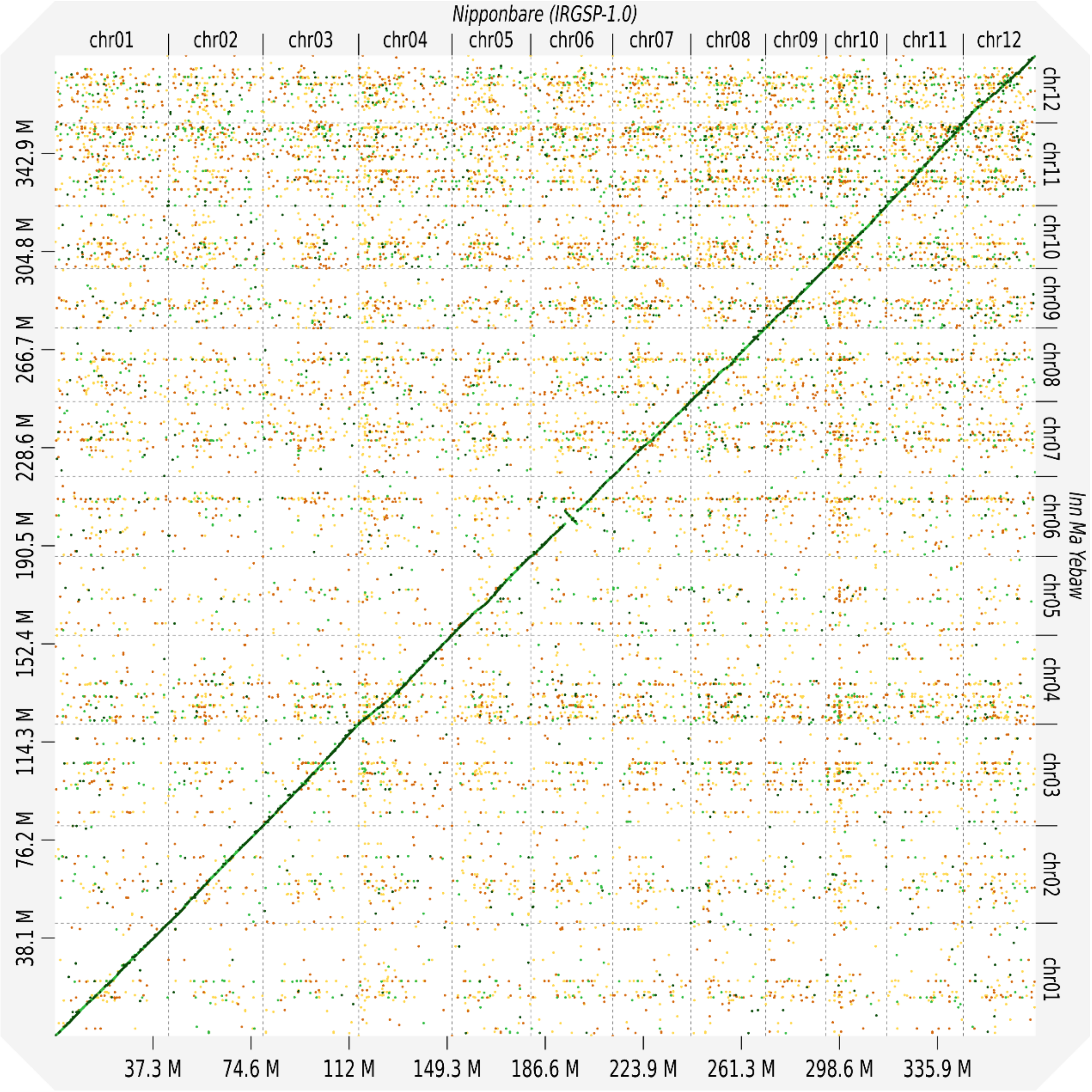

Supplemental Fig. 7. Collinearity of the IMY and NB genome assemblies.

The IMY genome assembly was aligned with the NB genome assembly, and then the alignment was visualized using D-GENIES. Each dot indicates an alignment of sequences in the NB genome assembly (x-axis) and the IMY genome assembly (y-axis). The dots plotted along the diagonal line indicate the sequences keeping collinearity in the genomes.

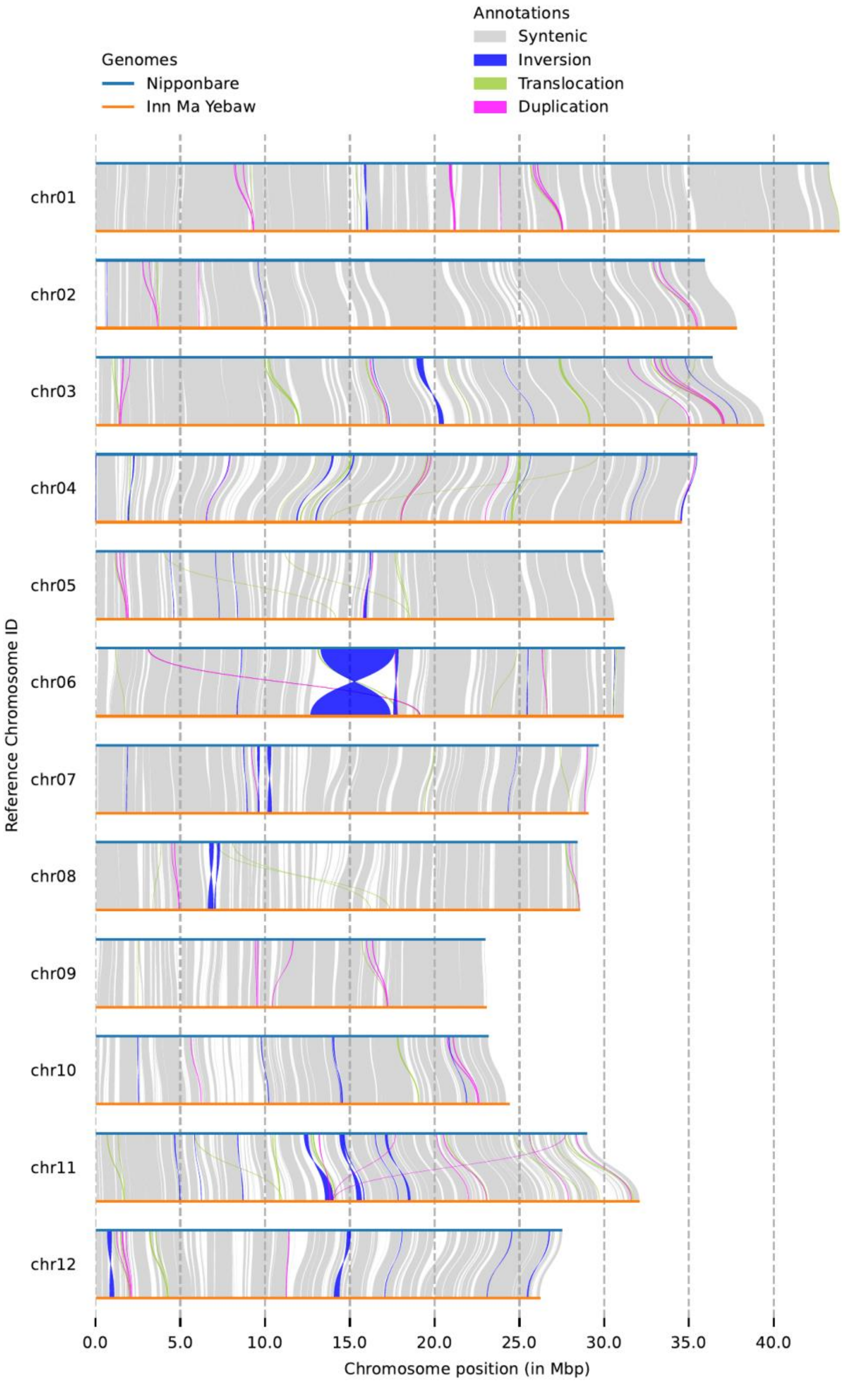

Supplemental Fig. 8. Structural variations between NB and IMY.

Syntenic regions, translocations, and duplications in each chromosome were visualized by colored ribbons, while inversions were shown as twisted colored ribbons. Regions with no ribbon indicate the genomic regions that were not aligned.

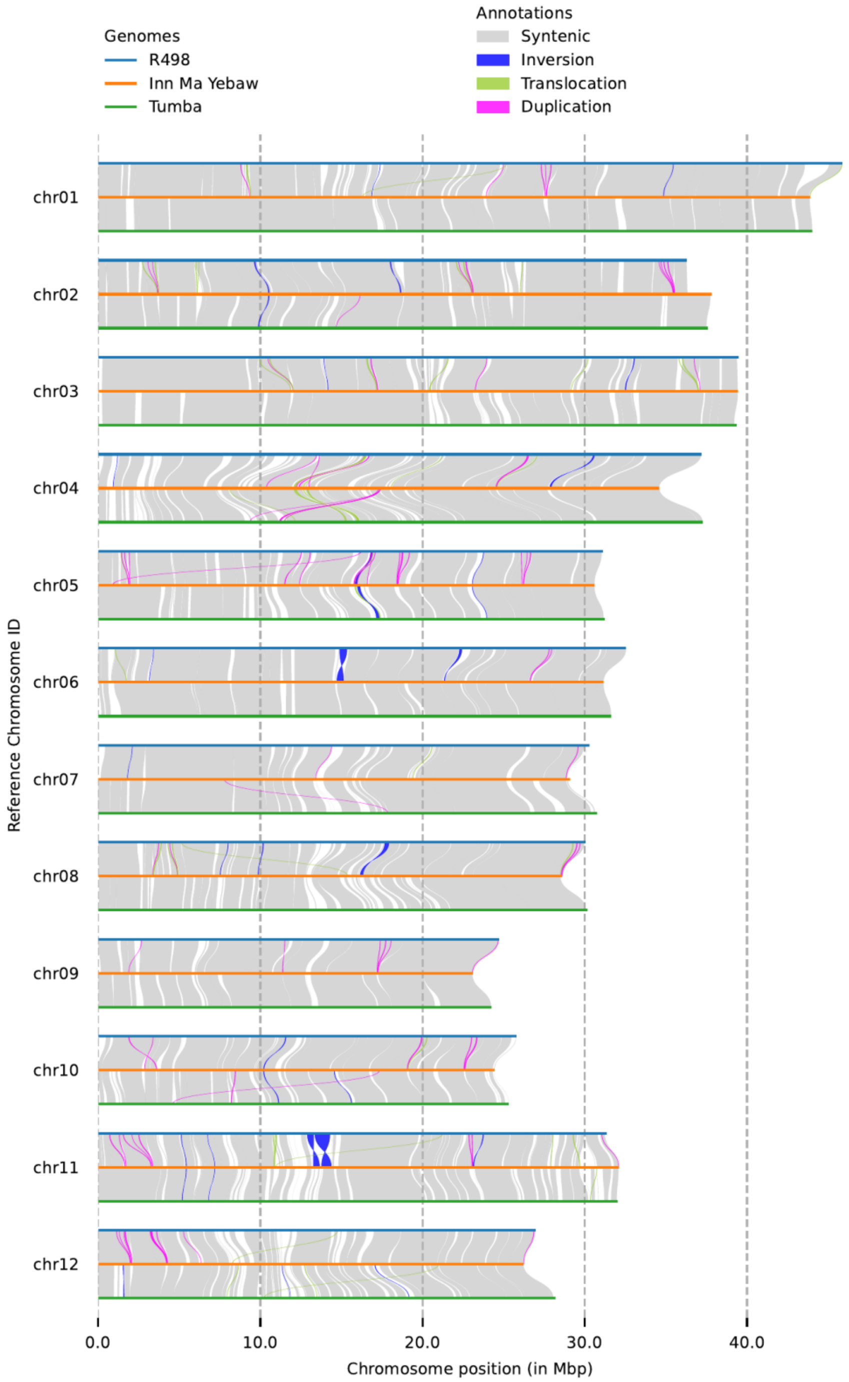

Supplemental Fig. 9. Structural variations between R498, Tumba, and IMY.
Syntenic regions, translocations, and duplications in each chromosome were visualized by colored ribbons, while inversions were shown as twisted colored ribbons. Regions with no ribbon indicate the genomic regions that were not aligned.

A

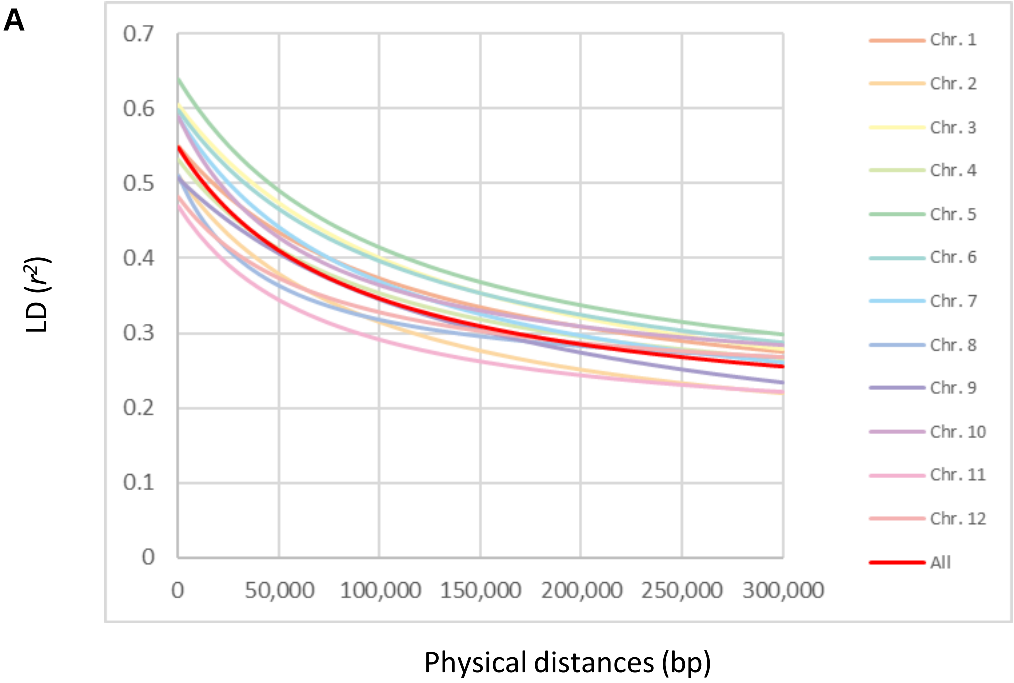

Supplemental Fig. 10. LD decay according to the physical distance (bp) between markers in each chromosome.

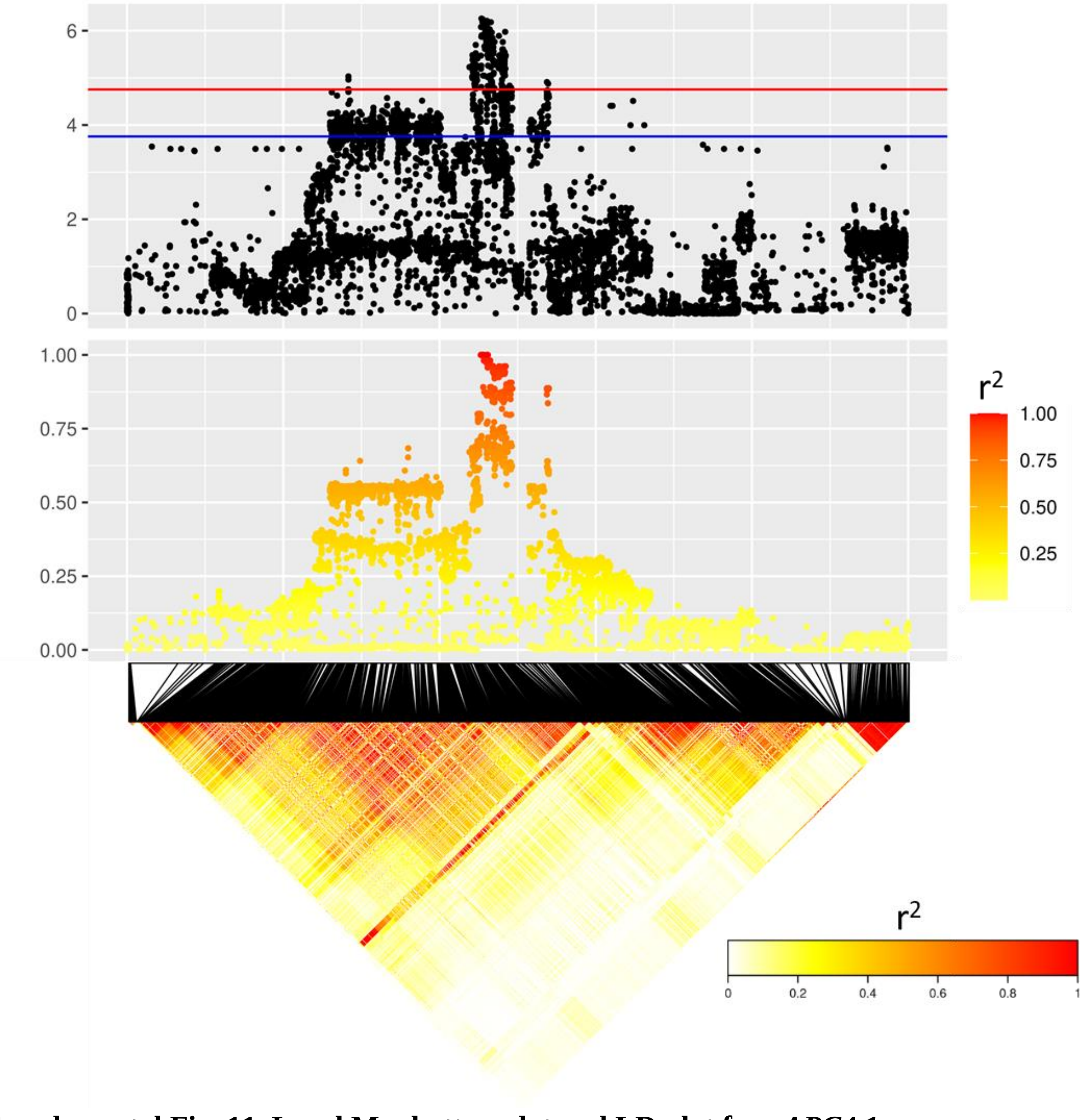

Supplemental Fig. 11. Local Manhattan plot and LD plot for *qAPC4.1*.

The top panel shows the local Manhattan plot around the association *qAPC4.1* detected in the GWAS using the IMY genome. The middle panel represents the linkage disequilibrium (r^2) of marker genotypes with the genotypes at the peak association marker. The LD block structure at this locus is visualized in the bottom panel. The red and blue horizontal lines in the top panel represent suggestive and significant threshold levels at FDR = 0.05 and 0.01, respectively.

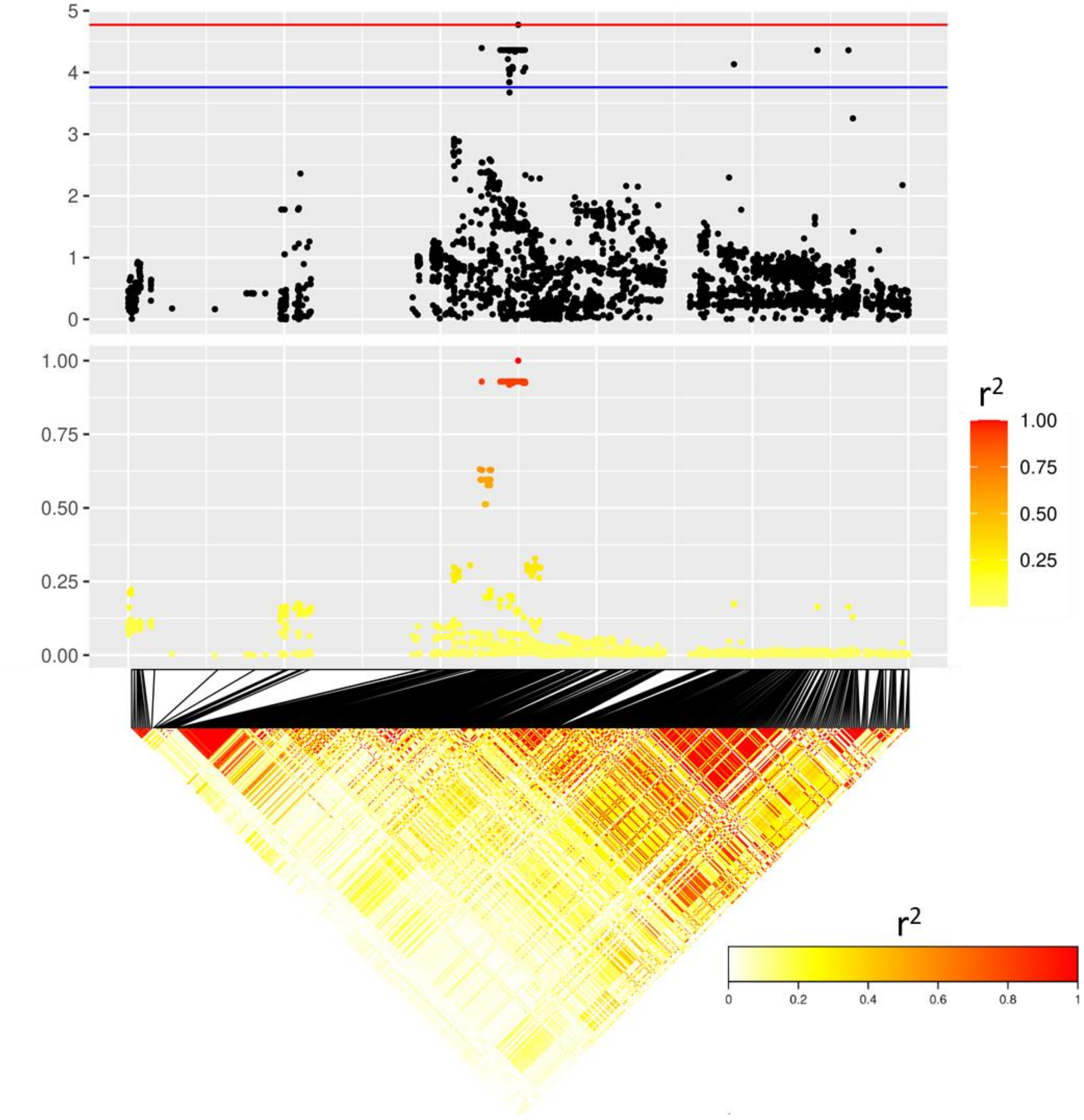

Supplemental Fig. 12. Local Manhattan plot and LD plot for *qAPC5.1*.

The top panel shows the local Manhattan plot around the association *qAPC5.1* detected in the GWAS using the IMY genome. The middle panel represents the linkage disequilibrium (r^2) of marker genotypes with the genotypes at the peak association marker. The LD block structure at this locus is visualized in the bottom panel. The red and blue horizontal lines in the top panel represent suggestive and significant threshold levels at FDR = 0.05 and 0.01, respectively.

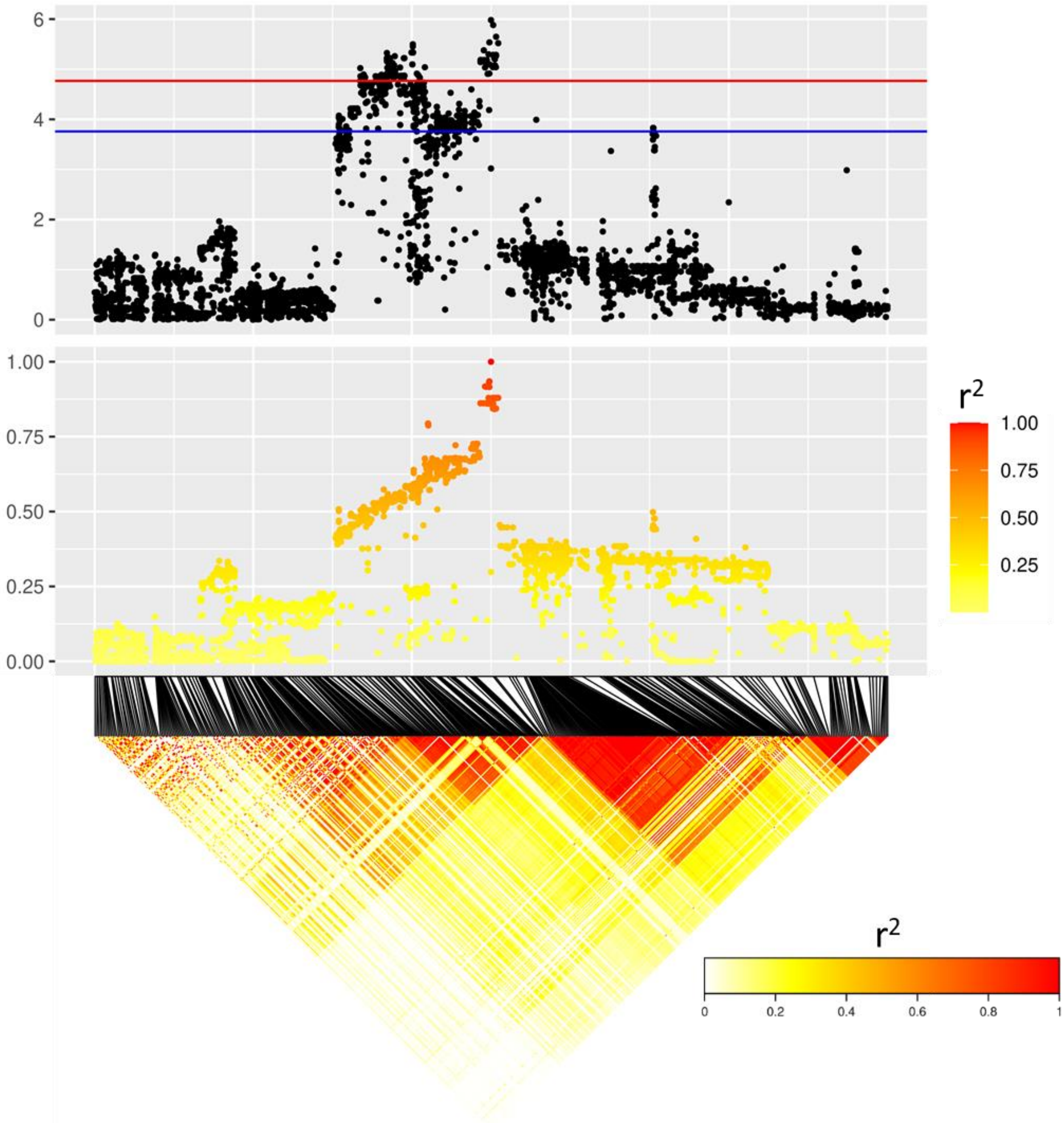

Supplemental Fig. 13. Local Manhattan plot and LD plot for *qAPC6.1*.

The top panel shows the local Manhattan plot around the association *qAPC6.1* detected in the GWAS using the IMY genome. The middle panel represents the linkage disequilibrium (r^2) of marker genotypes with the genotypes at the peak association marker. The LD block structure at this locus is visualized in the bottom panel. The red and blue horizontal lines in the top panel represent suggestive and significant threshold levels at FDR = 0.05 and 0.01, respectively.

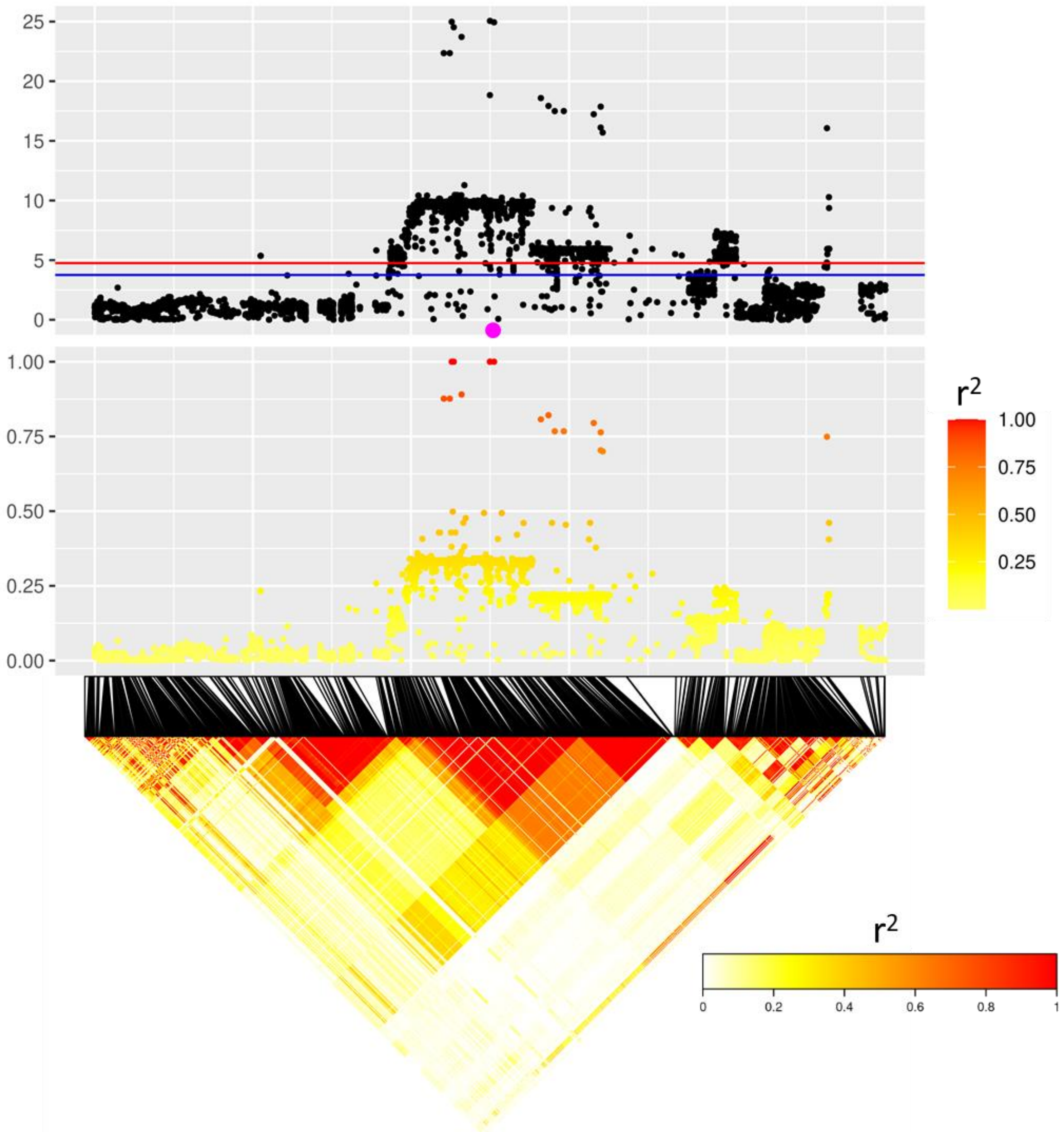

Supplemental Fig. 14. Local Manhattan plot and LD plot for *qAPC6.2*.
The top panel shows the local Manhattan plot around the association *qAPC6.2* detected in the GWAS using the IMY genome. The middle panel represents the linkage disequilibrium (r^2) of marker genotypes with the genotypes at the peak association marker. The LD block structure at this locus is visualized in the bottom panel. The red and blue horizontal lines in the top panel represent suggestive and significant threshold levels at FDR = 0.05 and 0.01, respectively. The magenta circle indicates the location of the *OsC1* ortholog in the IMY genome.

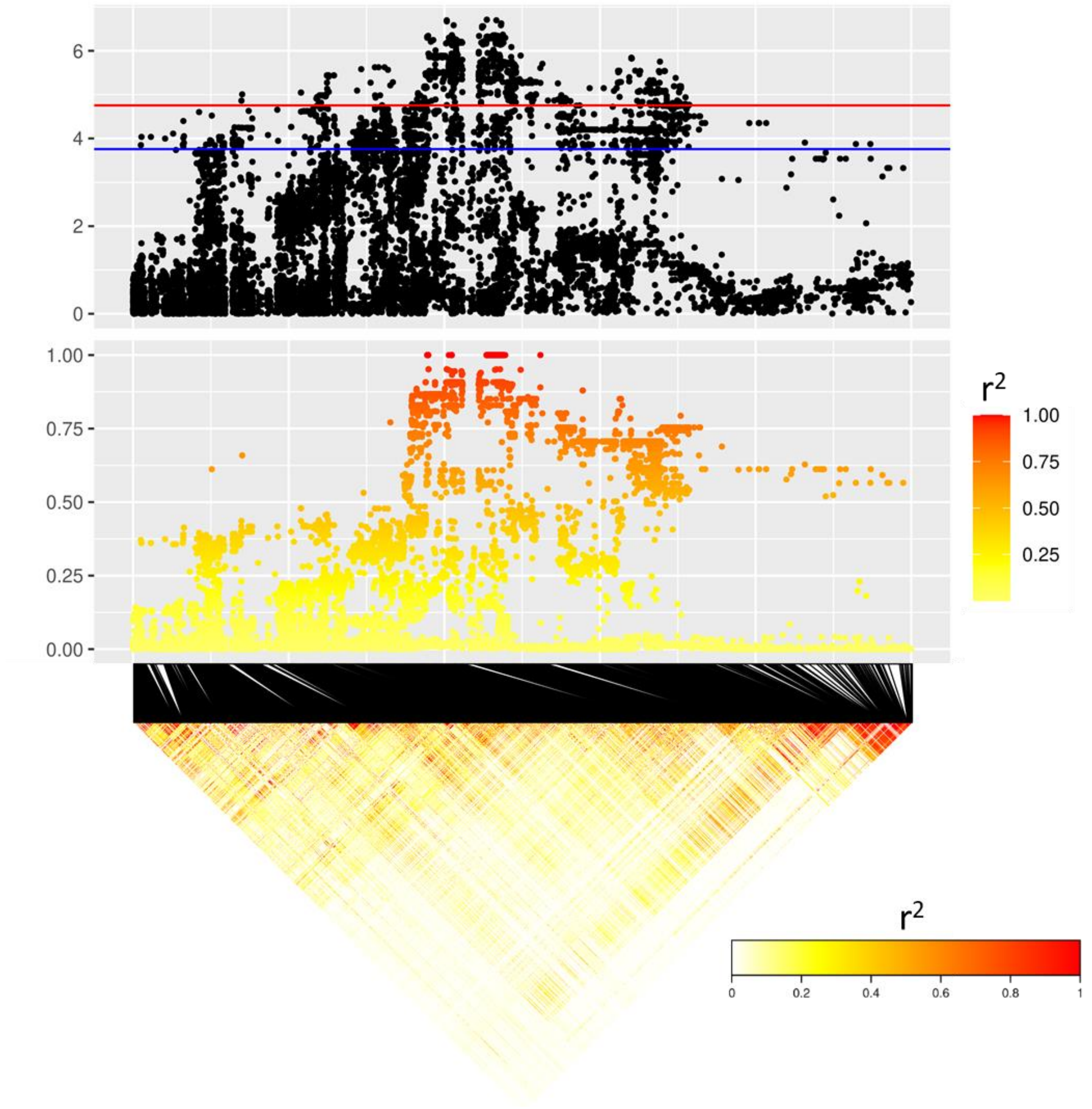

Supplemental Fig. 15. Local Manhattan plot and LD plot for *qAPC8.1*.

The top panel shows the local Manhattan plot around the association *qAPC8.1* detected in the GWAS using the IMY genome. The middle panel represents the linkage disequilibrium (r^2) of marker genotypes with the genotypes at the peak association marker. The LD block structure at this locus is visualized in the bottom panel. The red and blue horizontal lines in the top panel represent suggestive and significant threshold levels at FDR = 0.05 and 0.01, respectively.

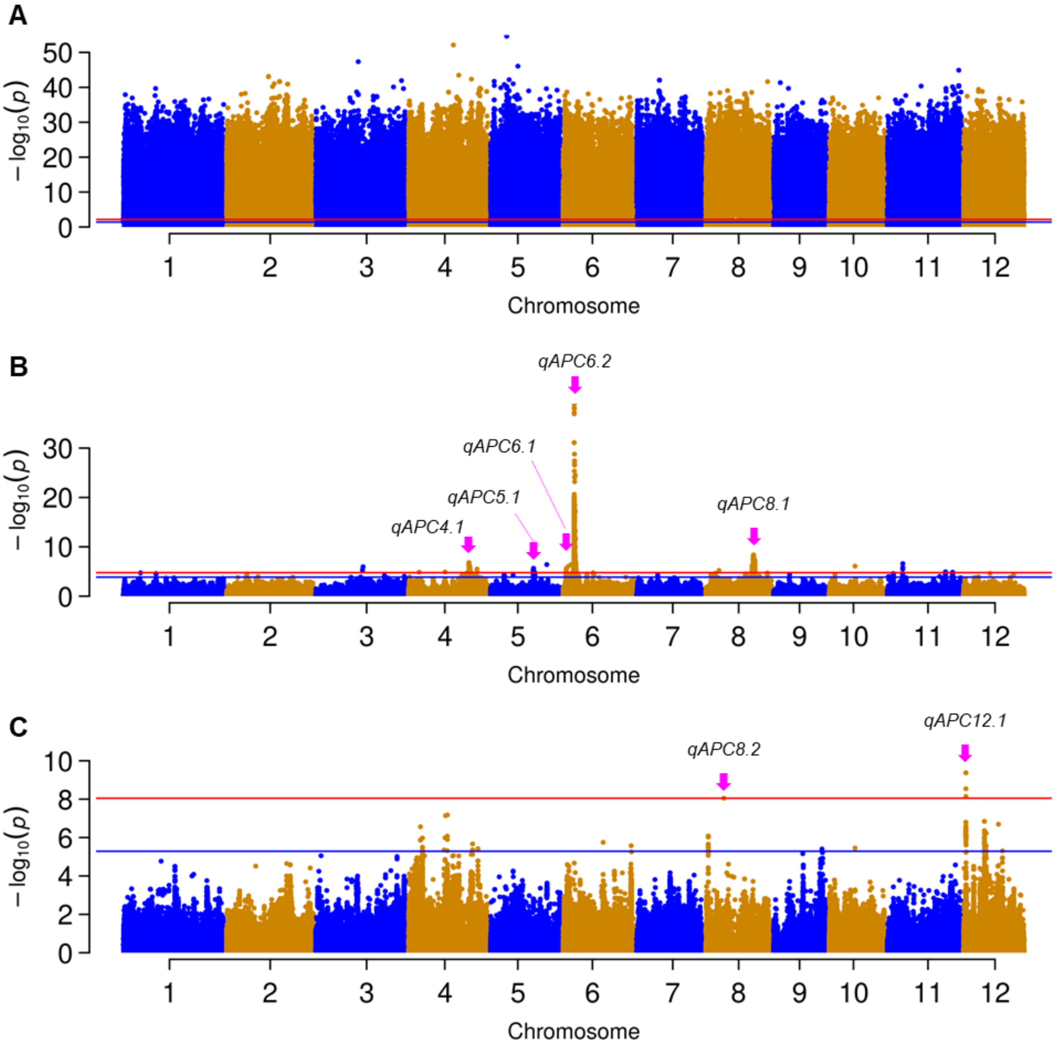

Supplemental Fig. 16. Manhattan plot of GWAS using the genotypes at *qAPC6.2* as a fixed effect.

The Manhattan plot shows the result of GWAS on apiculus pigmentation using the IMY genomes as a reference. The blue and red horizontal lines represent suggestive and significant threshold levels at FDR = 0.05 and 0.01, respectively. Significant QTL locations are represented by magenta arrows with labels indicating the QTL names. (A) If the logistic linear mixed model (LLMM) was used with including the genotypes at *qAPC6.2* as a fixed effect, severe inflation p-value inflations were observed throughout the genome. (B) Similar to the association tests using the LLMM without the fixed effect shown in Fig. 3A, the five significant QTLs as indicated by magenta arrows were observed even if the general linear mixed model (GLMM) was employed. (C) The association tests using the GLMM with the fixed effect eliminated the peaks at the five QTLs observed in panel A. Although *qAPC8.2* was not supported by multiple suggestive associations, we indicated it as a significant QTL.

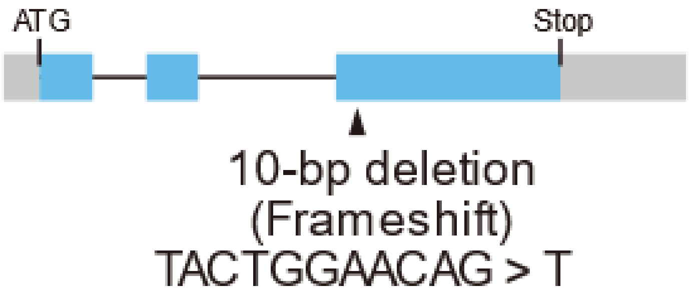

Supplemental Fig. 17. 10-bp deletion in the *OsC1* gene.
The schematic image represents the exon-intron structure of the *OsC1* gene. An arrowhead indicates the 10-bp deletion. The deletion in the coding sequence of the *OsC1* gene results in a frameshift leading to disruption of the function.

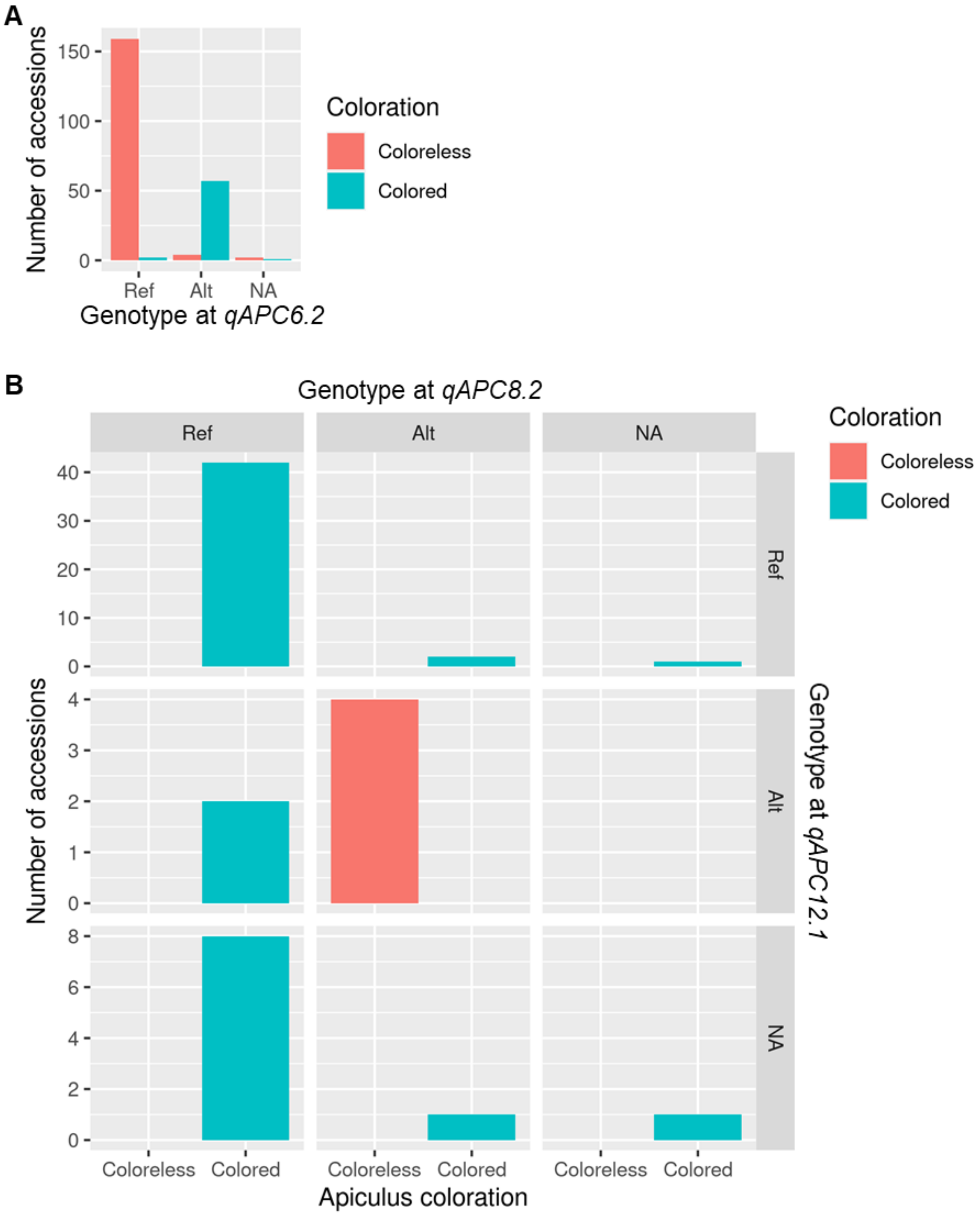

Supplemental Fig. 18. Phenotype by genotype plots for the candidate QTLs.

(A) The bar plot represents the apiculus coloration of the accessions possessing reference homozygous and alternative homozygous genotypes at the QTL *qAPC6.2*, which correlated with the presence or absence of a deletion in *OsC1*. The reference homozygote at *qAPC6.2* indicates the unfunctional *OsC1* leading to colorless apiculi. Four accessions showed colorless apiculi, while they had the functional *OsC1*. (B) The bar plots show the relationship between the apiculus coloration and the genotypes at *qAPC8.2* and *qAPC12.1*. Only the accessions having the functional *OsC1* were analyzed. When the genotypes at *qAPC8.2* and *qAPC12.1* were alternative homozygous, colorless apiculi were observed.
