# Supplementary material for: Development of genomic and genetic resources facilitating molecular genetic studies on untapped Myanmar rice germplasms
Source: Breed Sci. 2024 Mar 22;74(2):124–37. doi: 10.1270/jsbbs.23077 (PMC11442107; doi:10.1270/jsbbs.23077)
Supplement: Supplementary file 3 — Supplemental Text [file 74_124_s3.pdf]

doi: 10.1270/jsbbs.23077

Supplemental Text 1
The following scripts were used to perform the analyses described in the Materials and Methods section in the main text and Supplemental Methods.

- **Population structure analysis using *structure***

structure -i input_data.str -K 1 -o K1_output –D 1234
The number specified to the -K argument was changed from 1 to 10.
The input file "input_data.str" was created by the following codes.

```
# Filter for biallelic data
vcftools --gzvcf test.vcf.gz --max-alleles 2 --recode --stdout | bgzip -c > test2.vcf.gz

# Convert data to Hapmap format
~/tassel-5-standalone/run_pipeline.pl -fork1 -vcf test2.vcf.gz -homozygous -export data3 -exportType Hapmap -runfork1
# The output file will be named data3.hmp.txt.

# Download hmp2structure.pl
wget https://raw.githubusercontent.com/qikushu/stat/master/hmp2structure.pl

# Convert Hapmap data to STRUCTURE format
less data3.hmp.txt | perl hmp2structure.pl > input_data.str

# Determine the number of markers required for the STRUCTURE software
less input_data.str | awk -F'¥t' '{print NF; exit}'

# Determine the number of plants required for the STRUCTURE software
echo $(( $(wc -l < input_data.str ) - 2 ))
```

- **NJ tree construction for MIDP**

```
# Load and convert the input vcf file
vcf <- read.vcfR("filtered_variant.vcf.gz")
gl_vcf <- vcfR2genlight(vcf)

# Construct the NJ tree
tree <- nj(dist(as.matrix(gl_vcf)))
f <- function(x) nj(dist(as.matrix(x)))

# Bootstrapping
bootstrap <- boot.phylo(tr, gl_vcf, f, quiet = FALSE, B=100, tree=TRUE)
clad <- prop.clades(tree, bootstrap$tree, rooted = TRUE)
boot <- prop.clades(tree, bootstrap$tree)
tree$node.label <- boot
write.tree(tree, file = "tree.txt", append = FALSE, digits = 10, tree.names = FALSE)
```

The output file "tree.txt" was processed and the tree was visualized in the FigTree software.

- **FALCON-unzip**

The following settings were written in a json file named General_config.json and supplied to FALCON-unzip.

"LA4Falcon_preload": false,
"avoid_text_file_busy": true,
"bestn": 12,
"dazcon": false,
"falcon_sense_greedy": false,
"falcon_sense_option": "--output-multi --min-idt 0.70 --min-cov 4 --max-n-read 200",
"falcon_sense_skip_contained": false,
"fc_ovlp_to_graph_option": " --min-len 1000",
"genome_size": "360000000",
"input_fofn": "input.fofn",
"input_type": "raw",
"length_cutoff": "1000",
"length_cutoff_pr": "1000",
"overlap_filtering_setting": "--max-diff 100 --max-cov 100 --min-cov 2",
"ovlp_DBdust_option": "",
"ovlp_DBsplit_option": "-x500 -s100",
"ovlp_HPCdaligner_option": "-v -B128 -M24",
"ovlp_daligner_option": "-k18 -e.93 -l1800 -h1024 -s100",
"pa_DBdust_option": "",
"pa_DBsplit_option": "-x500 -s100",
"pa_HPCTANmask_option": "",
"pa_HPCdaligner_option": "-v -B128 -M24",
"pa_REPmask_code": "1,300;10,150;100,100",
"pa_daligner_option": "-k14 -e0.70 -l1000 -h256 -w8 -s100",
"pa_dazcon_option": "-j 4 -x -l 500",
"pa_fasta_filter_option": "streamed-internal-median",
"pa_subsample_coverage": 0,
"pa_subsample_random_seed": 12345,
"pa_subsample_strategy": "random",
"seed_coverage": 20,
"skip_checks": false,
"target": "assembly",
"ver": "100"

The following settings were written in a json file named Unzip_config.json and also supplied to FALCON-unzip.

"input_bam_fofn": "input_bam.fofn",
"input_fofn": "input.fofn",
"polish_include_zmw_all_subreads": true,
"polish_use_blasr": false,
"polish_vc_ignore_error": false

- **FALCON-unzip**

fc_run.py General_config.json
fc_unzip.py Unzip_config.json

- **Pilon**

bwa mem falcon_imy_contigs.fasta imy_srhot_reads_1.fq.gz imy_srhot_reads_2.fq.gz > imy_sr2contigs.bam

java -jar ~/tools/pilon/pilon-1.23.jar --genome falcon_imy_contigs.fasta --frags imy_sr2contigs.bam --output falcon_imy_pilon.fasta --outdir ./pilon_out

- **RaGOO**

ragoo.py falcon_imy_contigs.fasta tumba_genome.fa.gz -m minimap2

- **Comparative Annotation Toolkit (CAT)**

hisat2 --dta -k 3 -p 20 -x imy_genome.fa -1 tumba_rnaseq_R1.fq -2 tumba_rnaseq_R2.fq | samtools sort -O BAM > tumba_rnaseq.bam

samtools index tumba_rnaseq.bam

luigi --module cat RunCat --hal plants.hal --ref-genome=nip
--target-genomes='("imy",)' --config=cat.config --workers=1 --maxCores 10
--work-dir workdir --out-dir catout --local-scheduler --augustus
--augustus-species=rice --augustus-cgp --binary-mode singularity --batchSystem singleMachine

The following text shows the content of cat.config.
[ANNOTATION]
nip=nipponbare_gene_models.gff

[BAM]
imy=tumba_rnaseq.bam

- **genBlast**

genblast -p genblastg -q nipponbare_genes.prot.fa -t imy_genome.fa -g T -r 1 -o genbalst_output -gff -cdna -pro

- **InterProScan**

interproscan.sh -i imy_proteins.fa -b imy_proteins_interpro_output -f tsv -goterms

- **eggNOG**

emapper.py -i imy_proteins.fa -o imy_eggnog_output --tax_scope Streptophyta

- **RepeatMasker**

RepeatMasker imy_genome.fa -excln -s -html -gff -xsmall -no_is -species rice -dir imy_repmask_output

- **BLAST search for CentO**

makeblastdb -in imy_genome.fa -dbtype nucl

blastn -task blastn -db imy_genome.fa -query cento.fa > cento_blast_output

- **QTUAST**

quast imy_genome.fa -o imy_quast_output -e --large -k -f -s

- **BUSCO**

busco -m genome -i iyb_genome.fa -o imy_busco_output -l embryophyta_odb10

- **LAI calculation**

gt suffixerator -db imy_genome.fa -indexname imy_genome.fa -tis -suf -lcp -des -ssp -sds -dna

gt ltrharvest -index imy_genome.fa -minlenltr 100 -maxlenltr 7000 -mintsd 4 -maxtsd 6 -motif TGCA -motifmis 1 -similar 90 -vic 10 -seed 20 -seqids yes > harvest.scn

ltr_finder -D 15000 -d 1000 -L 7000 -l 100 -p 20 -C -M 0.9 imy_genome.fa > finder.scn

gt ltrharvest -index imy_genome.fa -minlenltr 100 -maxlenltr 7000 -mintsd 4 -maxtsd 6 -similar 85 -vic 10 -seed 20 -seqids yes > harvest.nonTGCA.scn

LTR_retriever -genome imy_genome.fa -infinder finder.scn -inharvest harvest.scn -nonTGCA harvest.nonTGCA.scn

- **Merqury**

meryl threads=16 memory=62g k=19 count output imy_1.meryl imy_short_read_R1.fq.gz

meryl threads=16 memory=62g k=19 count output imy_2.meryl imy_short_read_R2.fq.gz

meryl threads=16 memory=62g union-sum output imy.meryl imy_*.meryl

merqury.sh imy.meryl imy_genome.fa imy_clr > imy.log

- **D-GENIES**

The GUI version of D-GENIES was used in this study with default settings.

- **SyRI**

```
minimap2 -t 10 -ax asm5 --eqx nipponbare_genome.fa imy_genome.fa > out.sam

syri -F S -c out.sam -r nipponbare_genome.fa -q imy_genome.fa
```

- **GATK for phylogenetic classification of IMY**

```
bwa mem falcon_imy_contigs.fasta imy_srhot_reads_1.fq.gz
imy_srhot_reads_2.fq.gz | samtools view -Sb | samtools sort -o imy.bam
samtools index imy.bam

java -jar picard.jar MarkDuplicates I=imy.bam O=imy_dupmarked.bam
M=imy_dupmarked.metrics READ_NAME_REGEX=null

gatk --java-options "-Xmx50g" HaplotypeCaller -R nipponbare_genome.fa -I
imy_dupmarked.bam -O imy_variants.vcf.gz
```

- **R script for phylogenetic classification of IMY**

```
library(SNPRelate)
# variant_info.vcf contains variant information of IMY and 3KRG
snpgdsVCF2GDS(“variant_info.vcf”, “variant_info.gds”)
gds <- snpgdsOpen(“variant_info.gds”)

# LD based SNP marker pruning
pruned_snp <- snpgdsLDpruning(gds, maf = 0.05, missing.rate = 0.05, ld.threshold =
0.2)

# IBS calculation and dissimilarity matrix construction for the clustering
ibs <- snpgdsIBS(gds, snp.id = unlist((pruned_snp))
dm <- ibs$ibs
dm <- as.dist(1 - dm)
hc <- hclust(dm)

# Tree visualization
library(ggtree)
ggtree(hc, branch.length='none', layout='circular')
```

- **RFMix**

```
rfmix -f imy_variant.vcf -r 3KRG_variant.vcf -m sample.map -g genetic.map -o
imy_rfmix_chr<chr>_ --chromosome=<chr>
```

The above code was repeatedly executed for all chromosomes with replacing <chr> with chromosome IDs (1-12)

- **LD calculation**

```
# Define functions
# Function to return the number of alleles
f <- function(x) {
  return(length(levels(factor(as.genotype.allele.count(x)))))
```

```
}

# Function to calculate LD
ld <- function(x, y) {
	g1 = genotype(as.genotype.allele.count(x))
	g2 = genotype(as.genotype.allele.count(y))
	ld.result = LD(g1, g2)
	return(abs(ld.result$r))
}

library(genetics)
library(vcfR)
library(doParallel)
options(mc.cores = detectCores(logical = FALSE))

# Load and convert the input vcf file
vcf <- read.vcfR("filtered_variant.vcf.gz")
gl_vcf <- vcfR2genlight(vcf)
mat_selected <- as.matrix(gl_vcf)

# Extract chromosome and position information from marker names
marker <- colnames(mat_selected)
marker_list <- strsplit(marker, "_")
chr <- sapply(marker_list, function(x) x[1])
pos <- as.numeric(sapply(marker_list, function(x) x[2]))

# Randomly select SNP markers
n <- length(pos)
midx <- rep(1:n)
nsample <- 500000
loc1 <- sample(n, nsample, replace = T)
loc2 <- sample(n, nsample, replace = T)

# Calculate the marker distances and filter out too-distant (>10 Mb) marker pairs
pos_diff <- abs(pos[loc1] - pos[loc2])
max_dist <- 10000000
cond <- 0 < pos_diff & pos_diff < max_dist
loc_a1 = loc1[cond]
loc_a2 = loc2[cond]
mat_a1 = mat_selected[, loc_a1]
mat_a2 = mat_selected[, loc_a2]

# Check for non-polymorphic loci
cond_b1 <- mclapply(1:ncol(mat_a1), function(i) {
  col <- mat_a1[, i]
  result <- f(col) == 2
  return(result)
})
cond_b1 <- reduce("|", cond_b1)
cond_b2 <- mclapply(1:ncol(mat_a2), function(i) {
```

```
    col <- mat_a2[, i]
    result <- f(col) == 2
    return(result)
})
cond_b2 <- Reduce("|", cond_b2)
cond_b <- cond_b1 & cond_b2
loc_b1 <- loc_a1[cond_b]
loc_b2 <- loc_a2[cond_b]
distance <- abs(pos[loc_b1] - pos[loc_b2])
df_b1 <- as.data.frame(mat_selected[, loc_b1])
df_b2 <- as.data.frame(mat_selected[, loc_b2])

# Calculate LD
r2 <- mcmapply(ld, df_b1, df_bB2)
outdf <- data.frame(distance, r2)

# Write the result to a text file
write.table(outdf, “pairwise_ld.txt”)

# Plot the result
plot(outdf$distance, outdf$r, pch = ".", xlim = c(0, max(distance)), ylim = c(0, 1))
```

Pairwise LD values were obtained for marker pairs in each chromosome.

- **LD decay over physical distance**

```
# Load the output file of the pairwise LD calculation
pairwise_ld <- read.table("pairwise_ld.txt", head=T)
subset_df <- pairwise_ld[pairwise_ld$distance <= 300000, ]
distance <- subset_df$distance
ld_r2 <- subset_df$r2
n <- length(distance)
fit <- nls(ld_r2 ~ ((r2high - r2low) / (1 + C * distance) + r2low),
           start = list(C = 0.00001, r2high = 1, r2low = 0.1),
           control = nls.control(maxiter = 100, warnOnly = TRUE))
sum_fit <- summary(fit)
param <- sum_fit$parameters
est_c <- param["C","Estimate"]
estr2high <- param["r2high","Estimate"]
estr2low <- param["r2low","Estimate"]
pred <- ((estr2high - estr2low) / (1 + est_c * distance) + estr2low)

# Plot the LD decay over pairwise marker distance
pdf("LDPlot.pdf")
plot(distance, ld_r2, pch=".", ylim=c(0,1))
par(new=T)
plot(distance, pred, pch="." , ylim=c(0,1), col="red")
dev.off()
```

LD decay was estimated for each chromosome.

- **GWAS**

```
# Define functions
IntersectGenoPheno <- function(gt.score.t, pheno, colposition) {
   lines_g <- rownames(gt.score.t)
   lines_p <- rownames(pheno)
   lines_common <- intersect(lines_g, lines_p)
   gt.score.wp <- gt.score.t[lines_common, ]
   pheno_col <- as.numeric(as.character(pheno[lines_common, colposition]))
     p <- data.frame(gid = rownames(gt.score.wp), y = pheno_col)
   return(list(gt.score.wp, p))
}

GetGenotype101code = function(gt) {
      gt.score <- matrix(NA, nrow(gt), ncol(gt))
      gt.score[gt == "0/0"] <- -1
      gt.score[gt == "0/1"] <- 0
      gt.score[gt == "1/1"] <- 1
      gt.score[gt == "1|1"] <- 1
      gt.score[gt == "1|0"] <- 0
      gt.score[gt == "0|0"] <- -1
      gt.score[gt == "0|1"] <- 0
      rownames(gt.score) <- rownames(gt)
      colnames(gt.score) <- colnames(gt)
      return(t(gt.score))
}

SelectPeak = function(score, peakinterval = 200000, signifthreshold = 7.3,
suggestthreshold = 5, criteria_num=40) {
       score_sig_max_out <- data.frame()
       peak_num <- 1
       maxpos <- max(score$pos)

       while (1) {
           # select the SNPs with more than -log10(p) = suggested threshold (5).
           if (sum(score$y > suggestthreshold) > 0) {
           numscorerow <- length(score$y)
           score_sig <- score[score$y > suggestthreshold,]
           # Select the peak
           highest_idx <- which.max(scoreSig$y)
           score_sig_max <- score_sig[highest_idx,]

           # counting SNPs on upstream and downstream of the highest SNPs.
           score_sig_max_pos <- score_sig_max$pos
           score_sig_max_pos_lower <- score_sig_max_pos - peakinterval
           score_sig_max_pos_upper <- score_sig_max_pos + peakinterval
           if (score_sig_max_pos_upper > maxpos) {
                score_sig_max_pos_upper <- maxpos
           }

           # Remove the peak SNPs from the dataframe
```

```
            score_wo_sig_max_pos = score_sig[-highest_idx,]

            # Define the index within the range and extract.
            inrange = score_wo_sig_max_pos$pos > score_sig_max_pos_lower &
                score_wo_sig_max_pos$pos < score_sig_max_pos_upper
            score_inrange = score_wo_sig_max_pos[inrange, ]
            # Counting of SNPs
            score_better = score_inrange[score_inrange$y > suggestthreshold, ]
            num_score_better = dim(score_better)[1]

            # If the number of SNPs in the range is more than the criteria_num
            if (num_score_better > criteria_num) {

                # Output the SNPs
                score_sig_max_memo <- data.frame(peak_num,
                                                 score_sig_max, "peak")
                colnames(score_sig_max_memo) <- c("peakNum","marker",
                                                  "chr","pos","y", "peak")
                score_sig_mid_memo <- data.frame(peak_num,
                                                 score_better, "notPeak")
                colnames(score_sig_mid_memo) <- c("peakNum","marker",
                                                  "chr","pos","y", "peak")
                 Peak_num <- peak_num + 1
                if (!exists("score_sig_max_out")) {
                    score_sig_max_out <- rbind(score_sig_max_memo,
                                               score_sig_mid_memo)
                } else {
                score_sig_max_out <- rbind(score_sig_max_out,
                                           score_sig_max_memo,
                                         score_sig_mid_memo)
                }
            }
                outrange <- !inrange
                score <- score_wo_sig_max_pos[outrange, ]
        } else {
            return(score_sig_max_out)
        }
        }
}

library(vcfR)
library(rrBLUP)

# Construct genomic relationship matrix
vcf <- read.vcfR("variant.vcf.gz")
pheno <- read.table("phenotype.tsv", row.names = 1)
# Extract genotype data from vcf
gt <- extract.gt(vcf)
gt1 = GetGenotype101code(gt)
geno_pheno_df <- IntersectGenoPheno(gt1, pheno, 1)
```

```
gt2 <- igeno_pheno_df[[1]]
# Calculate the genomic relationship matrix
amat <- A.mat(gt2, shrink = TRUE)

# Regression analysis
amat.m <- data.matrix(amat)
vcffix = fix(vcf)
chrom <- getCHROM(vcf)
pos <- getPOS(vcf)
p <- intersectedGenoPhenoList[[2]]
g <- data.frame(colnames(gt2),
                as.numeric(factor(chrom)), pos, t(gt2))
rownames(g) <- seq_len(nrow(g))
colnames(g) <- c("marker", "chrom", "pos", rownames(gt2))
gwa <- GWAS(p, g, K = amat, n.PC = 0, min.MAF = 0.05, plot = FALSE)
gwascore <- data.frame(gwa)
write.table(gwascore,"gwas_score.txt")

selected_gwa_score = SelectPeak(score = gwascore, peakinterval = 96521,
signifthreshold = 7.3, suggestthreshold = 5)
write.table(selected_gwa_score, "selected_gwas_score.txt")
```

The followings are the contents of files uploaded in the data repository.

FASTA files

imy_genome_chr.fa.gz: Inn Ma Yebaw reference genome sequence

imy_all_transcript.fa.gz: Inn Ma Yebaw predicted transcript sequences

imy_all_protein.fa.gz: Inn Ma Yebaw predicted protein sequences

imy_contigs.fa.gz: Inn Ma Yebaw all contig sequences subjected in the assembling of the Inn Ma Yebaw genome

GFF files

imy_genome_annotation.gff.gz: All gene models and annotations containing the following GFF files

imy_repeat.gff.gz: TEs and repeaet elements annotated by RepeatMasker

imy_contigs.gff.gz: Contig anchoring position in the IMY gneome

VCF files

MIDP_on_IMY_cleaned_snps.vcf.gz: Variant (SNPs) information of Myanmar indica diversity panel (MIDP) obtained by mapping short reads on the IMY genome assembly

MIDP_on_NB_cleaned_snps.vcf.gz: Variant (SNPs) information of Myanmar indica diversity panel (MIDP) obtained by mapping short reads on the Nipponbare genome assembly (IRGSP-1.0)

Files for population structure analysis

extraparams_for_STRUCTURE: extraparams for the STRUCTURE software

mainparams_for_STRUCTURE: mainparams for the STRUCTURE software

input_data_for_STRUCTURE.str: genotype data for the STRUCTURE software

Supplemental Text 2

Supplemental Methods

Genotyping and assembling a diversity panel

From the seed bank of the Department of Agricultural Research, Yezin, Myanmar, 610 representative accessions were selected based on phenotypes and geographical origins (Supplemental Table 1). These accessions included the representative Myanmar rice varieties Inn Ma YeBaw (IMY), Mote Soe Ma Kyway Kyay (MSMKK), and Paw San Hmwe (PSH), which are an elite lowland variety, an elite upland variety, and a premium aromatic variety, respectively. Since new varieties bred from an existing variety have been sometimes given the same variety name as the parental variety in Myanmar, Supplemental Table 1 contains redundant variety names.

Total genomic DNA was extracted from the representative accessions according to the method described by Dellaporta *et al*. (1983), with minor modifications. The genomic DNA was digested by two enzymes KpnI and MspI to construct a sequencing library for Illumina SBS technologies with minor modification (Poland *et al.* 2012). Sequencing was performed using the MiSeq platform (Illumina, San Diego, CA, USA). The obtained short-read sequences were processed using the GBS v2 pipeline implemented in the Tassel 5 software with default parameters (Bradbury *et al.* 2007) to conduct genotype calling. The single nucleotide polymorphism (SNP) markers were filtered based on the minor allele frequency (> 0.05) and missing rate (≤ 0.5). To reduce the redundancy in the genotype information, only one SNP was selected in every 1 kbp stretch of the genome sequence. The SNP filtering described above was also conducted using Tassel 5.

The variant information obtained using GBS was subjected to principal component analysis (PCA) to visualize the population structure of the 610 Myanmar rice accessions (Supplemental Fig. 1). For the PCA, the variant information was converted to the R/genlight object using the vcfR2genlight() function in R/vcfR version 1.13.0 (Knaus and Grünwald 2017) and subjected to principal component analysis using the glPca() function of R/adegenet version 2.1.8 (Jombart and Ahmed 2011). The first and second principal components (PC1 and PC2) separated the accessions into three major clusters, Cluster I, Cluster M, and Cluster P, which include IMY, MSMKK, and PSH, respectively (Supplemental Fig. 1A). The majority of the accessions were plotted in Cluster I. Based on these observations, the accessions were screened based on the following conditions; $-11 < PC1 < 0$, $-5 < PC2 < 5$, $-11 < PC3 < 10$, $-7.5 < PC4 < 10$, $-3 < PC5 < 1$ (Supplemental Fig. 1E). After adjusting the number of accessions to fit our research capacity, a diversity panel designated as MIDP consisting of the 250 screened accessions was assembled as listed in Supplemental Table 2.

Sequencing the IMY genome

The whole-genome resequencing of the IMY genome was conducted as follows. First, 60-80 mg each of young etiolated leaves, which had not emerged from subtending leaf sheathes, were sampled from well-grown IMY plants in the paddy field in the Department of Agricultural Research, Yezin, Myanmar. The leaf samples had been kept in a -80°C freezer until extraction. Each of the sampled leaves was put in a 2.0 mL microtube with two 3-mm stainless beads and frozen in liquid nitrogen. The leaf sample was crushed using a Multi Beads Shocker (Yasui-Kikai, Osaka, Japan) at 1600 rpm for two cycles of a 20-second shake. The crushed leaf sample was well mixed

with 600 μl of extraction buffer (1% polyvinylpyrrolidone 40, 1% sodium metabisulfite, 0.5 M sodium chloride, 100 mM pH 8.0 Tris, 50 mM M EDTA, 1.25% sodium dodecyl sulfate, 100 mg/ml RNase A). The sample-mixed solution was incubated for 10 min at 65°C followed by a 10-min incubation on ice after mixing 200 μl of 5M potassium acetate. Insolubilized contaminants were precipitated by centrifugation for 10 min at 5000 g. An equal volume of isopropyl alcohol was then added to the sample solution to insolubilize DNA. After mixing the solution for 5 min gently, an appeared white DNA precipitate was hooked up and transferred into 70% ethanol for rinsing. The DNA precipitate was dried up once and then dissolved in 60 μl of Buffer EB (QIAGEN). We subjected 20 batches of 60-80 mg leaf samples to DNA extraction in total and put the extracted DNA solutions together into three tubes. The three tubes of the extracted DNA solution were shipped to BGI Genomics, Shenzhen, China, for sequencing. The sequencing using PacBio Sequel was conducted on three SMRT cells. The sequencing using BGI DNBSEQ-500 was also conducted by BGI genomics using genomic DNA of IMY extracted by the standard CTAB method (Doyle and Doyle 1987).

Phylogenetic classification of IMY

Since we had no additional data for linking contigs into chromosome-scale assembly like Hi-C and optical mapping (Lam *et al.* 2012, van Berkum *et al.* 2010), a high-quality reference genome was required to scaffold contigs into pseudomolecules. Therefore, we first conducted a phylogenetic analysis to identify the most optimal reference genome assembly for scaffolding the IMY genome assembly. A previous study using over 3000 rice varieties (3KRG) demonstrated that the world's rice varieties could be classified into nine sub-groups including four *indica* (XI-1A, XI-1B,

XI-2, XI-3), three *japonica* (GJ-trp, GJ-sbtrp, GJ-tmp), circum-Aus (cA) and circum-Busmati (cB) (Wang *et al.* 2018). Thus, we analyzed the phylogenetic relationship of IMY and 3KRG based on their variants. First, we obtained variant information by aligning 332,005,644 short reads obtained from IMY by DNBSEQ-500 as described in the section above. The short reads were aligned on the NB genome assembly IRGSP-1.0 (Kawahara *et al.* 2013, Sakai *et al.* 2013), which was obtained from RAP-DB (https://rapdb.dna.affrc.go.jp), using the read aligner BWA-MEM (https://doi.org/10.48550/arXiv.1303.3997). The resultant alignment information was then processed via GATK to call variants (McKenna *et al.* 2010, van der Auwera *et al.* 2020). The default settings were used for both BWA-MEM and GATK. As the next step, the 404k SNPs dataset of 3KRG was downloaded from Rice SNP-Seek Database (https://snp-seek.irri.org) (Mansueto *et al.* 2017). The 404k SNPs dataset was merged with the SNPs dataset obtained by the short-read alignment described above. To reduce redundancy in genotype information, less correlated SNPs were selected using the snpgdsLDpruning function of the R package SNPRelate with the settings “maf = 0.05, missing.rate = 0.05, ld.threshold = 0.2” (Zheng *et al.* 2012). An Identity-By-State (IBS) distance matrix was computed from the pruned SNPs dataset using the snpgdsIBS function of SNPRelate and used to construct a phylogenetic tree to visualize the sub-group classification of IMY using the hclust function implemented in the stats package of R. The tree was visualized using the ggtree package (Yu *et al.* 2017). In the phylogenetic tree, IMY was grouped in the XI-3 clade (Supplemental Fig. 2 and 3). The same SNPs dataset was also subjected to the local-ancestry inference of the IMY genome using RFMix (Maples *et al.* 2013). The SNPs dataset was split into two VCF files: one included the data for IMY only and another included 3K-RG. The local-ancestry inference was performed using the

VCF file of IMY as a query and another as reference with the default settings. The local ancestry analysis using RFMix also indicated that IMY is purely XI-3 (Supplemental Table 4). Since IMY was classified in the XI-3 subgroup, we selected as a reference the genome assembly of Tumba, which is only one XI-3 variety in the previously published 33 high-quality genome assemblies (Qin *et al.* 2021).

Generating a circle plot for the IMY genome assembly

To overview the assembly, we generated a circle plot with the following tracks. The contig alignment track was generated based on the output files from RaGOO that provide alignment information of contigs anchored in the resultant pseudomolecules. Transposable Element density (TE density) in each 500-kb bin was calculated based on the output generated by RepeatMasker as described above. The total number of nucleotides identified as transposable elements in each bin was divided by 500,000 to obtain a density in each bin. The TE density was visualized as a line plot. The number of genes in each 500-kb bin was also calculated by counting up gene loci modeled and plotted as a heatmap to visualize the uneven distribution of genes in the genome. The proportion of G and C nucleotides in each 500-kb bin of the genome sequence was calculated as GC content. To detect single nucleotide polymorphisms (SNPs), insertions (INSs), and deletions (DELs) between the genome assemblies of IMY and NB, we compared the genome assemblies using SyRI (Goel *et al.* 2019). Since SyRI requires whole genome alignment information for a given pair of genomes, we aligned the genome sequences using minimap2 with the settings “-ax asm5 --eqx” (Li 2018). We used the default settings for running SyRI. Each of the three variant types was extracted from the SyRI’s output and summarized as a separate variant call format (VCF) file (Danecek *et al.* 2011). To visualize densities of SNPs, INSs, and

DELs found between the genome assemblies of IMY and NB, the number of each variant type in each 100-kb bin was counted based on the output from the SyRI that we obtained as described in the previous section. The variant densities were represented as bar plots for the three variant types, respectively. Putative centromeric regions were identified by BLAST search with the rice satellite repeat sequence CentO (GenBank accession: AF058902) as a query (Dong *et al.* 1998). If a 500-kb bin had one or more BLAST hits, we defined the bin as a centromeric region. The centromeric bins were highlighted in yellow on the ideogram track. The data for the circle plot was prepared using R with the following packages: Biostrings, rBLAST, BSgenome, GenomicRanges, and data.table (Dowle and Srinivasan 2023, Hahsler and Nagar 2019, Lawrence *et al.* 2013, Pagès 2022, Pagès *et al.* 2022, R Core Team 2023). The circle plot was generated using the circle plot drawing tool Circos (Krzywinski *et al.* 2009).

Literature Cited

van der Auwera, G., B. O'Connor and an O. M. Company (2020) Safari, Genomics in the cloud: Using docker, GATK, and WDL in Terra.

Danecek, P., A. Auton, G. Abecasis, C. A. Albers, E. Banks, M.A. DePristo, R.E. Handsaker, G. Lunter, G.T. Marth, S.T. Sherry *et al.* (2011) The variant call format and VCFtools. Bioinformatics 27: 2156.

Dellaporta, S.L., J. Wood and J.B. Hicks (1983) A plant DNA minipreparation: Version II. Plant Mol Biol Report 1: 19–21.

Dowle, M. and A. Srinivasan (2023) data.table: Extension of `data.frame`. R package version 1.14.8, https://CRAN.R-project.org/package=data.table.

Doyle, J. J. and J. L. Doyle (1987) A rapid DNA isolation procedure for small quantities of fresh leaf tissue. Phytochem Bull 19: 11–15.

Hahsler, M. and A. Nagar (2019) rBLAST: R Interface for the Basic Local Alignment Search Tool. R package version 0.99.2, https://github.com/mhahsler/rBLAST.

Krzywinski, M., J. Schein, I. Birol, J. Connors, R. Gascoyne, D. Horsman, S.J. Jones and M.A. Marra (2019) Circos: An information aesthetic for comparative genomics. Genome Res 19: 1639–1645.

Lawrence, M., W. Huber, H. Pagès, P. Aboyoun, M. Carlson, R. Gentleman, M.T Morgan and V.J. Carey (2013) Software for computing and annotating genomic ranges. PLoS Comput Biol 9: e1003118.

Li, H. (2018) Minimap2: Pairwise alignment for nucleotide sequences. Bioinformatics 34: 3094–3100.

Mansueto, L., R.R. Fuentes, F.N. Borja, J. Detras, J.M. Abrio-Santos, D. Chebotarov, M. Sanciangco, K. Palis, D. Copetti, A. Poliakov *et al.* (2017) Rice SNP-seek database update: new SNPs, indels, and queries. Nucleic Acids Res 45: D1075–D1081.

Maples, B.K., S. Gravel, E.E. Kenny and C.D. Bustamante (2013) RFMix: A discriminative modeling approach for rapid and robust local-ancestry inference. Am J Hum Genet 93: 278.

McKenna, A., M. Hanna, E. Banks, A. Sivachenko, K. Cibulskis, A. Kernytsky, K. Garimella, D. Altshuler, S. Gabriel, M. Daly *et al.* (2010) The Genome Analysis Toolkit: A MapReduce framework for analyzing next-generation DNA sequencing data. Genome Res 20: 1297–1303.

Pagès, H. (2022) BSgenome: Software infrastructure for efficient representation of full genomes and their SNPs. https://doi.org/10.18129/B9.bioc.BSgenome.

Pagès, H., P. Aboyoun, R. Gentleman and S. DebRoy (2022) Biostrings: Efficient manipulation of biological strings. https://doi.org/10.18129/B9.bioc.Biostrings.

Poland, J.A., P.J. Brown, M.E. Sorrells and J.-L. Jannink (2012) Development of high-density genetic maps for Barley and Wheat using a novel two-enzyme genotyping-by-sequencing approach. PLoS One 7: e32253.

Yu, G., D. Smith, H. Zhu, Y. Guan and T.T. Lam (2017) ggtree: an R package for visualization and annotation of phylogenetic trees with their covariates and other associated data. Methods Ecol Evol 8: 28-36.

Zheng, X., D. Levine, J. Shen, S. M. Gogarten, C. Laurie and B.S. Weir (2012) A high-performance computing toolset for relatedness and principal component analysis of SNP data. Bioinformatics 28: 3326–3328.
